# PDOL-Based Solid Electrolyte Toward Practical Application: Opportunities and Challenges

**DOI:** 10.1007/s40820-024-01354-z

**Published:** 2024-02-21

**Authors:** Hua Yang, Maoxiang Jing, Li Wang, Hong Xu, Xiaohong Yan, Xiangming He

**Affiliations:** 1 Institute for Advanced Materials, School of Materials Science and Engineering, Jiangsu University, Zhenjiang, 212013 People’s Republic of China; 2 Institute of Nuclear and New Energy Technology, Tsinghua University, Beijing, 100084 People’s Republic of China

**Keywords:** Poly(1,3-dioxolane), Solid electrolyte, Polymerization mechanism, Composite electrolyte, Practical application

## Abstract

Polymer solid-state lithium batteries (SSLB) are regarded as a promising energy storage technology to meet growing demand due to their high energy density and safety. Ion conductivity, interface stability and battery assembly process are still the main challenges to hurdle the commercialization of SSLB. As the main component of SSLB, poly(1,3-dioxolane) (PDOL)-based solid polymer electrolytes polymerized in-situ are becoming a promising candidate solid electrolyte, for their high ion conductivity at room temperature, good battery electrochemical performances, and simple assembly process. This review analyzes opportunities and challenges of PDOL electrolytes toward practical application for polymer SSLB. The focuses include exploring the polymerization mechanism of DOL, the performance of PDOL composite electrolytes, and the application of PDOL. Furthermore, we provide a perspective on future research directions that need to be emphasized for commercialization of PDOL-based electrolytes in SSLB. The exploration of these schemes facilitates a comprehensive and profound understanding of PDOL-based polymer electrolyte and provides new research ideas to boost them toward practical application in solid-state batteries.

## Highlights

- The poly(1,3-dioxolane) (PDOL) electrolyte demonstrates promising potential for practical application due to its advantages in in-situ polymerization process, high ionic conductivity, and long cycle life.
- This review focuses on the polymerization mechanism, composite innovation, and application of PDOL electrolytes.
- This review provides a comprehensive summary of the challenges associated with the PDOL electrolyte and makes forward-looking recommendations.

## Graphical abstract

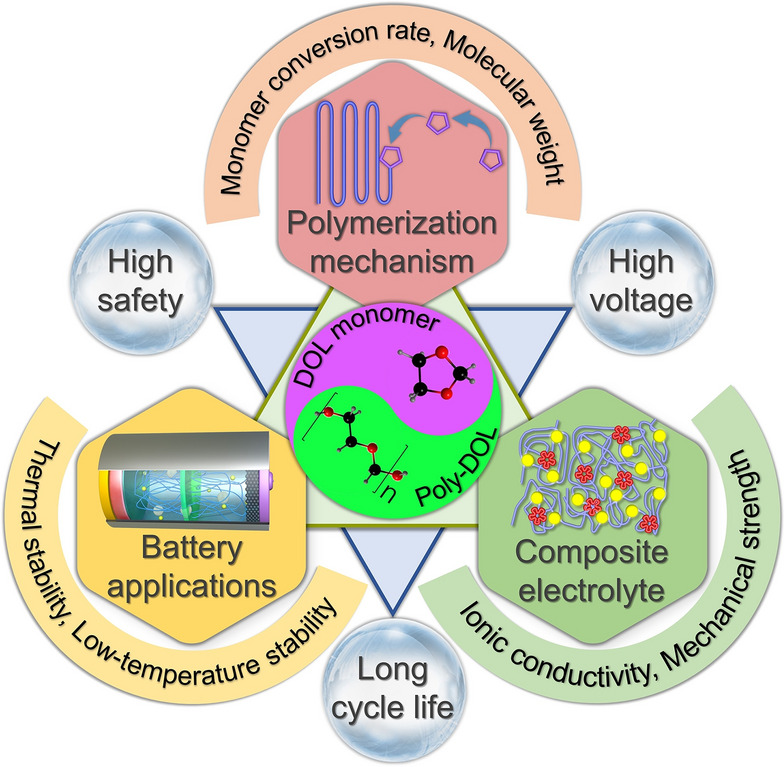

## Introduction

The mass production of lithium-ion batteries (LIB) has profoundly transformed our daily lives over the past few decades, with continuous performance improvement and widespread use [1]. In recent years, the development of electric vehicles has driven the demand for key performance indicators of lithium-ion batteries, including energy density, safety, and service life [2], to a new height. Solid-state lithium batteries (SSLB) employ a solid-state ion conductor to replace conventional liquid electrolytes. With the advantages of less flammable electrolytes and more stable electrochemical properties, they are considered one of the most competitive solutions to break through the performance bottleneck of traditional liquid lithium-ion batteries in the future [3]. The core solid electrolyte (SE) of SSLB has been continuously optimized and developed into two major categories: solid polymer electrolytes (SPE) and solid inorganic electrolytes (SIE). SPE has promising application prospects due to its lower cost, superior interface contact, and ease of production and processing [3–6]. SPE currently comprises solid electrolyte systems that mainly consist of materials such as polyethylene oxide (PEO) [7–11], polyvinylidene fluoride (PVDF) [12–18], pyrolytic polyacrylonitrile (PAN) [19–21], and poly(vinylidene fluoride-co-hexafluoropropylene) (PVDF-HFP) [22–24], poly(methyl methacrylate) (PMMA) [23–29], poly(vinyl chloride) (PVC) [30], which have relatively good electrochemical performance owing to their respective advantages. PEO is one of the earliest and most extensively investigated SPEs, exhibiting numerous advantages such as cost-effectiveness, excellent thermal stability, and remarkable flexibility. However, it suffers from challenges related to low ionic conductivity and environmentally detrimental preparation processes. Therefore, SPEs encounter research bottlenecks due to limitations such as narrow electrochemical stability window, low room temperature ionic conductivity, poor infiltration into porous electrodes, and other defects. SIE has attracted significant attention due to its higher room temperature ionic conductivity, wider electrochemical stability window, higher mechanical strength, and excellent non-flammability. Research on SIE is centered around two main categories: oxides (Li_7_La_3_Zr_2_O_12_, Li_1.3_Al_0.3_Ti_1.7_(PO_4_)_3_, Li_6_BaLa_2_Ta_2_O_12_, etc.) [31–33] and sulfides (Li_3.25_Ge_0.25_P_0.75_S_4_, Li_3.4_Si_0.4_P_0.6_S_4_, etc.) [34, 35]. SIE with no leakage and high safety is an ideal electrolyte material for achieving truly solid-state lithium-ion batteries. However, the practical application of SIE poses more apparent challenges. SIE with higher stiffness introduces more interface problem. Its more complex structure results in higher raw material costs, production costs, and more stringent requirements for preparation environment. These limitations have slowed down the development of SSLB, especially in response to the increasing demand for higher electrochemical performance from LIB in terminal products [36–38].

**Fig. 1:**
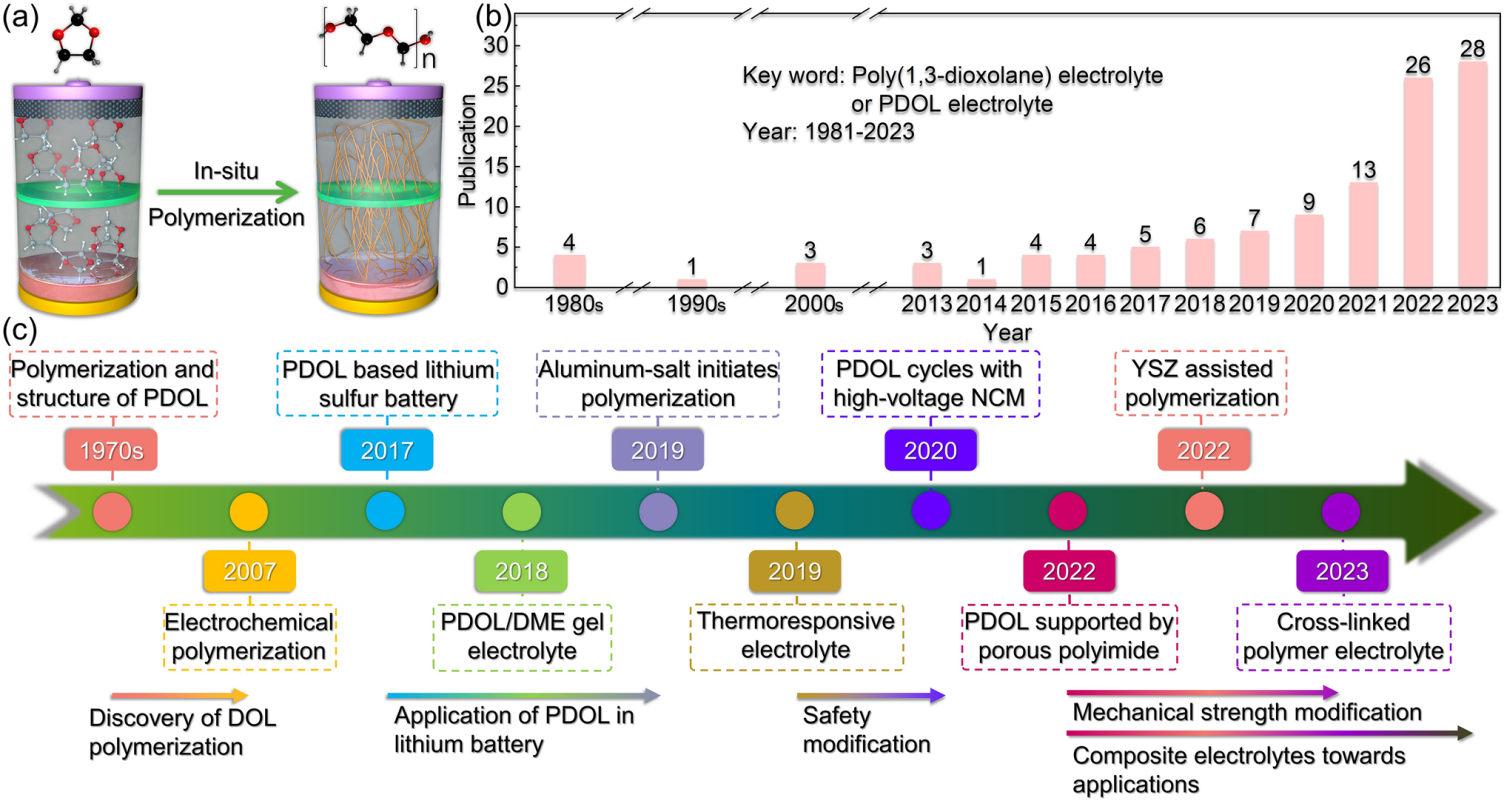
Overview of research progress on PDOL polymer. **a** Schematic diagram of in-situ polymerization of DOL monomer inside the battery. **b** Number of publications related to PDOL electrolyte in each year (Keyword: Poly(1,3-dioxolane) electrolyte or PDOL electrolyte; Data from Web of Science). **c** Development process of PDOL polymer: The polymerization of DOL and the structure of PDOL began to be studied (1968–1972) [60–62], Current is used to initiate DOL polymerization (2007) [63], PDOL inhibits the shuttle effect of polysulfides in lithium-sulfur batteries (2017) [56], PDOL/DME gel electrolyte enabled stable cycle with LiFePO_4_ cathode (2018) [58], Initiating DOL polymerization with anhydrous participation of trace aluminum salts (2019) [64], PDOL/PLAS thermoresponsive electrolyte prevents thermal runaway (2019) [65], Realizing normal cycling of PDOL initiated with the assistance of AlF_3_ and high-voltage NCM cathode (2020) [66], Porous polyimide supported PDOL enhances the mechanical strength of the electrolyte (2022) [67], PDOL added by YSZ enables long life cycling at high voltage (2022) [68], PDOL-based cross-linked electrolyte enhances the stability of lithium metal (2023) [69]

In recent years, in-situ polymerization has emerged as a promising method for the preparation of SE, offering significant advantages in terms of processing and cost [12, 39–46]. Even though in-situ polymerization has the potential for problems such as non-uniform polymerization and unpredictable polymerization rates. This approach involves the conversion of liquid monomers into solid polymers under specific conditions, which effectively addresses the issue of poor interface contact between solid-state electrolytes and solid-state electrodes (Fig. 1a) [38, 39, 47, 48]. Out of these, poly(1,3-dioxolane) (PDOL), which is a polymerization product of 1,3-dioxolane (DOL), shows promising potential for application in lithium-ion batteries. This is attributed to its excellent stability to lithium metal, good antioxidant stability, and facile polymerization method [49].

The remarkable stability of PDOL in relation to lithium metal renders it a suitable candidate for deployment as an artificial solid electrolyte interphase (SEI) and has been extensively studied [50, 51]. Additionally, as an SEI, PDOL can guide the uniform deposition of Li^+^ ions and reduce interfacial side reactions [52]. Especially in lithium-sulfur batteries, protecting the lithium metal anode is one of the core strategies to enhance its performance [53–56], the in-situ solidified PDOL can effectively suppress the shuttling of polysulfides and extend their cycling life [57–59].

Although there is limited research on PDOL as an electrolyte, the number of related publications is increasing rapidly recently (Fig. 1b) (based on the Web of Science database), particularly in high-voltage lithium battery systems moving toward practical applications. Given the significant potential application prospects of PDOL polymer electrolytes, this paper provides a categorization and summary of the current research progress of PDOL polymer electrolytes that are still in the early stage of development (Fig. 1c). The paper also summarizes the effects of various modification approaches on polymer properties, and provides a comprehensive understanding of the PDOL polymer electrolyte system for new researchers. Finally, the paper offers reasonable perspectives for future research on the practical application of PDOL in high-voltage lithium batteries.

## Fundamental Mechanism of DOL Polymerization

Research on the polymerization of DOL can be traced back to 1967 when P. H. Plesch and P. H. Westermann [70] successfully polymerized DOL in dichloromethane using anhydrous perchloric acid as a catalyst. The resulting polymer comprised alternating “formaldehyde” and “epoxyethane” units. Yuya Yamashi [60] found that water decreases the rate of DOL polymerization when catalyzed by triethylammonium boron tetrafluoride. The study also introduced the novel concept that the induction period preceding polymerization is inversely proportional to the initial catalyst concentration. Moreover, the study revealed a linear correlation between the degree of polymerization and conversion, suggesting that the polymerization reaction proceeds even in the absence of water. This study offers a valuable reference for comprehending the internal polymerization dynamics of in-situ polymerization reactions. Furthermore, the copolymerization of DOL and the crystal structure of PDOL were subsequently investigated, providing further theoretical insight into the polymerization of DOL [61, 62].

**Fig. 2:**
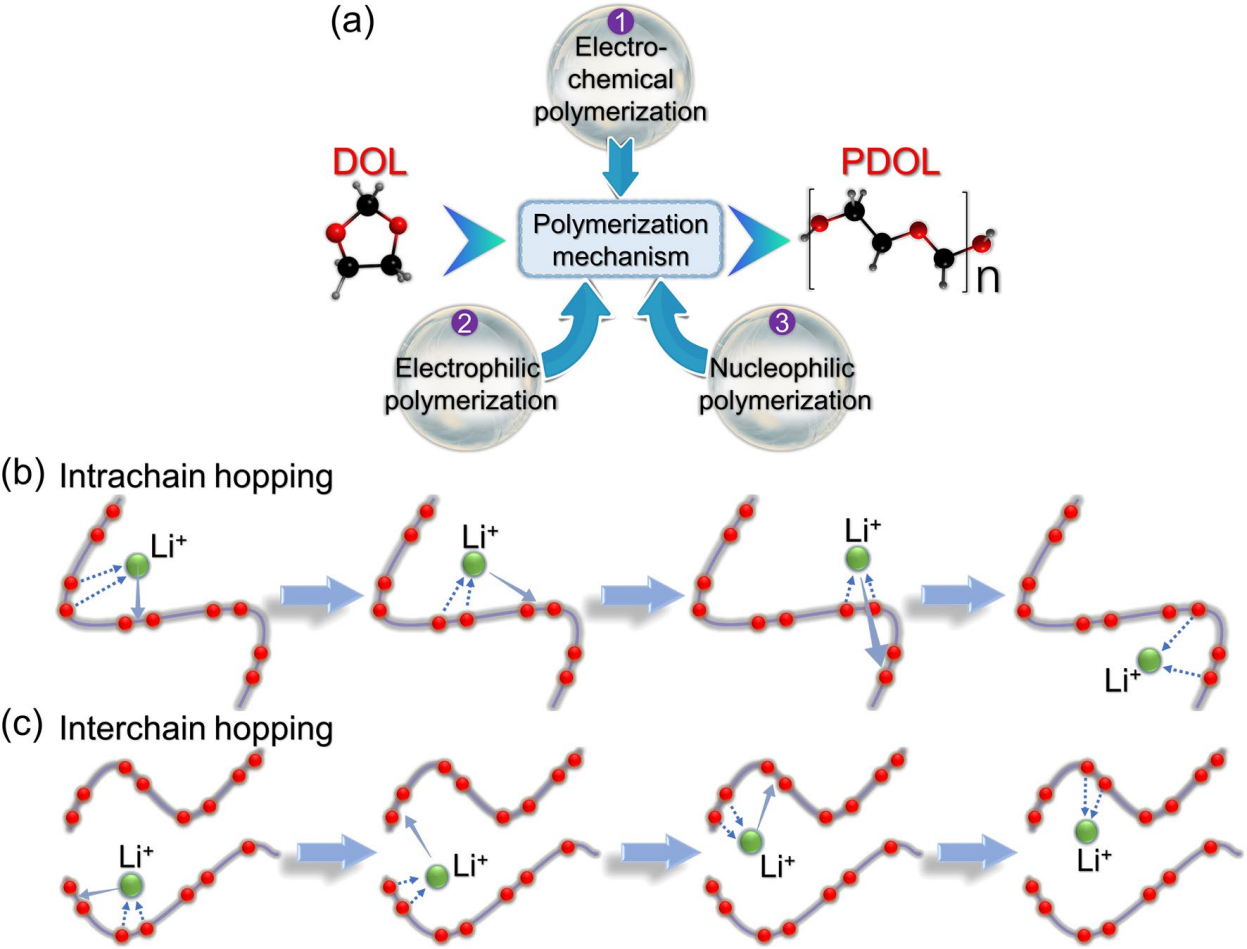
Polymerization mechanism and Li^+^ conduction mechanism of DOL. **a** Three polymerization mechanisms of DOL: electrochemical polymerization, electrophilic polymerization, and nucleophilic polymerization [71]. Schematic diagram of **b** intrachain conduction mechanism and **c** interchain conduction mechanism for Li.^+^ in PDOL [72]

Subsequently, Aurbach Doron [71] employed a range of techniques such as surface-sensitive Fourier transform infrared spectroscopy (FT-IR), scanning electron microscopy (SEM), X-ray microanalysis, linear sweep voltammetry (LSV), and other methods to investigate the polymerization mechanism of DOL. It was found that electrochemical treatment and contact with Al_2_O_3_ or LiAsF_6_ can form O(–CH_2_CH_2_–O–CH_2_–)_*x*_O-type polyether substances, which leads to the growth of PDOL chains. This study also provided comprehensive insight into the polymerization mechanism of DOL, encompassing electrochemical polymerization, electrophilic polymerization, and nucleophilic polymerization (Fig. 2a). These findings offer valuable guidance for selecting initiators for in situ polymerization of DOL in future investigations.

The function of polar atoms in facilitating Li^+^ conduction in polymers highlights the significance of their content in the polymer chain. The PDOL polymer chain contains a high proportion of polar oxygen atoms, amounting to 43.2 wt%, which is considerably higher than that of PEO (36.4 wt%). This represents a key factor contributing to the generally higher ionic conductivity of PDOL compared to PEO at room temperature.

The ion conductivity of solid polymer electrolytes is typically facilitated by the complexation and decomplexation of Li^+^ ions with polar atoms [72]. As with PEO segments, the plausible modes of Li^+^ conduction in PDOL encompass intrachain conduction (Fig. 2b) and interchain conduction (Fig. 2c). In short-range conduction, Li^+^ continuously complexes and decomplexes with oxygen atoms on the same PDOL chain under the action of an electric field to achieve migration, that is, intrachain conduction (Fig. 2c). Long-range conduction necessitates interchain conduction, wherein Li^+^ ions decomplex from an oxygen atom on one PDOL chain under the influence of an electric field and migrate to another chain to complex with its oxygen atom, thereby completing interchain conduction (Fig. 2d).

### Electrochemical Polymerization of DOL

**Fig. 3:**
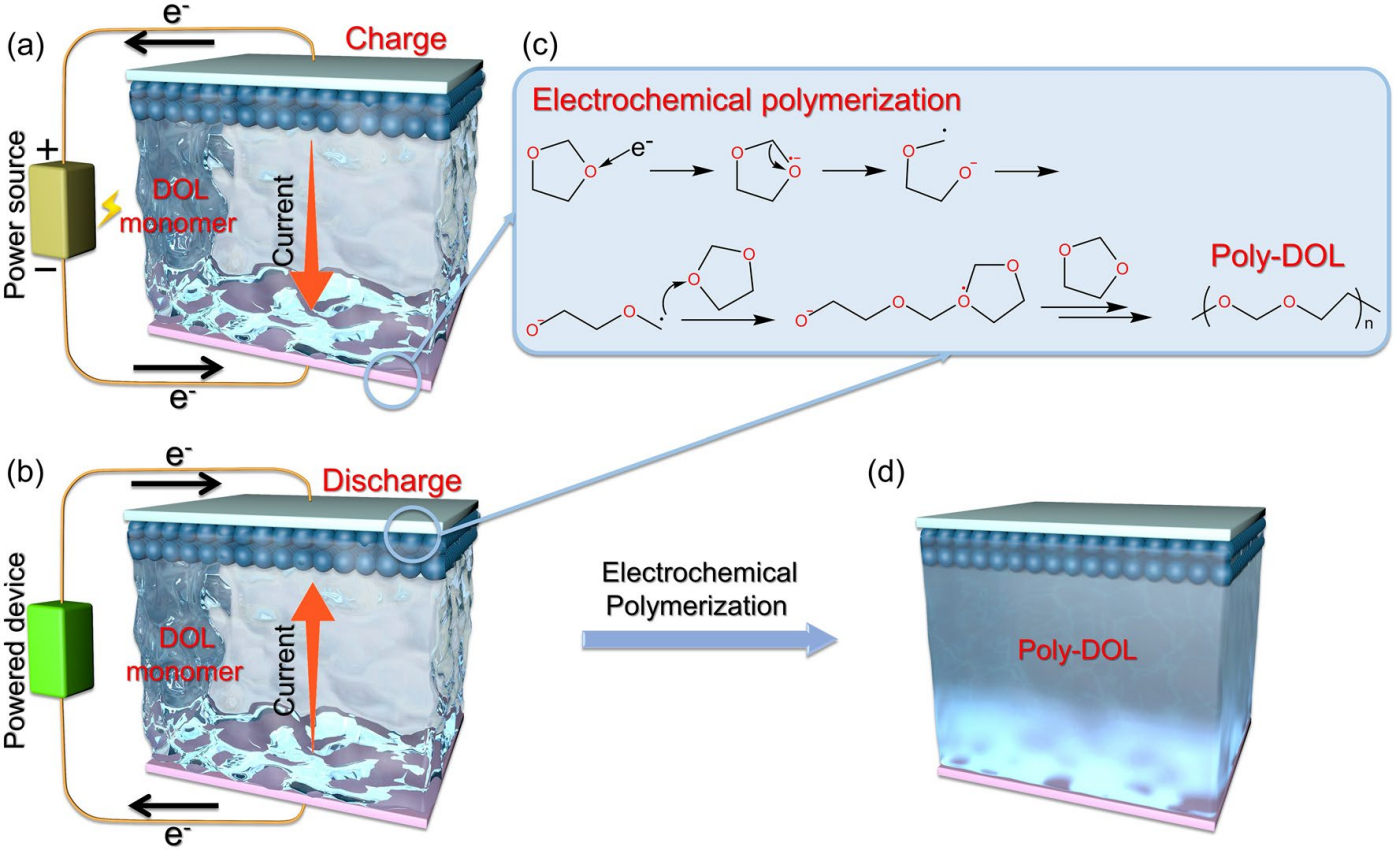
Applications of current-initiated DOL polymerization. Schematic diagram of electronic and current movements during **a** charging and **b** discharging processes. **c** Electrochemical polymerization mechanism of DOL. **d** Battery state after current-initiated DOL polymerization [45]

Despite the long-standing use of DOL monomers as electrolyte solvents for lithium-ion batteries, they are highly susceptible to decomposition and consequent battery failure at high voltage [73]. Therefore, early DOL-based solvents were often only used in low-voltage battery systems, such as lithium-sulfur batteries. In contrast, as the polymerization product of DOL, PDOL exhibits a markedly improved tolerance to high voltages. Hui Zhan [63] applied an appropriate voltage to in-situ polymerize DOL, resulting in a specific current value that enabled the utilization of DOL-based electrolytes in high voltage LiCoO_2_ cathode batteries (Fig. 3a, b). This is a typical example of DOL polymerization caused by electrochemical polymerization. When the oxygen atom on the DOL ring is attacked by electrons, the C-O bond is broken. The oxygen atom at the end of the broken chain segment is negatively charged and generates -CH_2_ radical. And free radicals will combine with the oxygen of DOL monomers to initiate the next polymerization reaction (Fig. 3c). The authors employed an electrolyte comprising 1M LiTFSI in DOL/DME (1,2-dimethoxyethane) at a weight ratio of 2:1 and assembled cells with LiCoO_2_ and lithium metal. The cells were subsequently cycled at current densities of 20, 50, 100, 200, and 500 mA g^−1^. The results indicated that DOL remained inactive and the cycling performance rapidly deteriorated at low current densities (20, 50 mA g^−1^), whereas the cell exhibited sustained stable cycling over 100 mA g^−1^. The cyclic voltammetry (CV) curve of the cell corroborated that the polymerization process was irreversible and did not influence the electrochemical performance of LiCoO_2_. The author believes that these surprising performance improvements are due to current-initiated DOL polymerization forming PDOL (Fig. 3d). The prolonged cycle life demonstrated that the DOL polymerization induced by high current possesses higher dependability in high-voltage battery systems.

### Electrophilic Polymerization of DOL

**Fig. 4:**
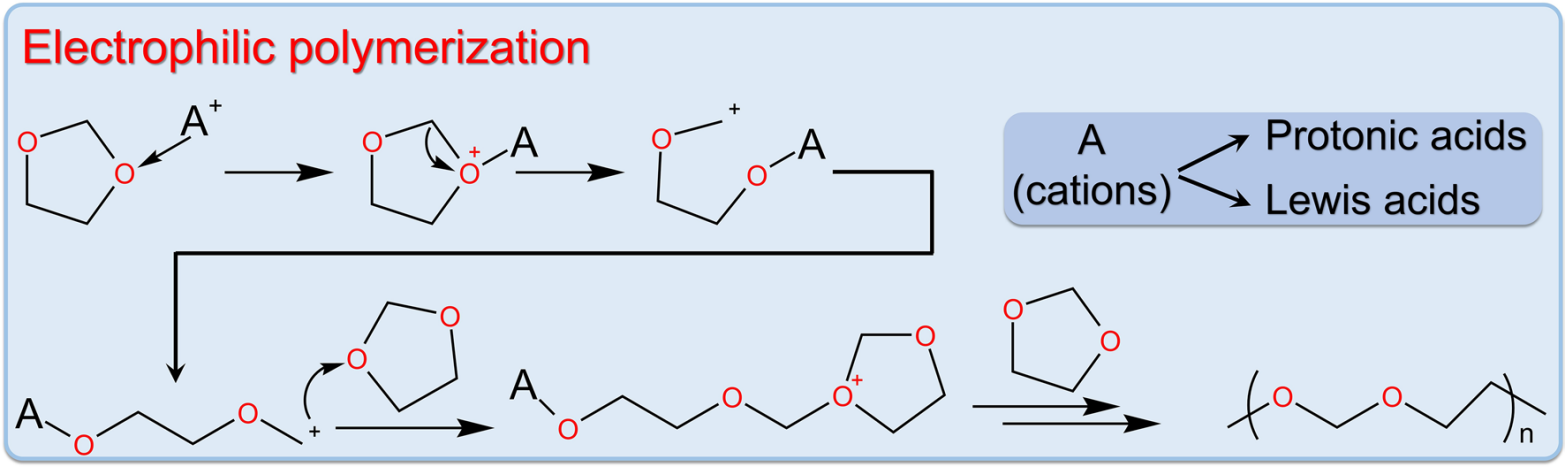
Electrophilic polymerization mechanism of DOL [71]

DOL polymerization often involves the loss or donation of electrons by the oxygen atom on the ring. Consequently, the electrophilic species are ideal initiators for DOL polymerization, and these electrophilic catalytic reactions can quickly and effectively promote the ring-opening polymerization of DOL. These electrophilic catalysts belong to a category of DOL ring-opening initiators, typically in the form of cations, including protonic acids and Lewis acids [71, 74]. They all initiate ring-opening polymerization by capturing electrons from the oxygen atoms on the DOL ring (Fig. 4).

We have systematically categorized the initiators mentioned in the current literatures, which primarily include lithium salts, aluminum salts, tin salts, other salts, acidified materials, and organic materials (Table 1). Lithium salt initiators often react with trace amounts of water to generate protonic acids for initiating DOL polymerization, while acidified materials rely on the presence of H^+^ on the surface of the material to initiate polymerization. Metal salts such as aluminum and zinc salts typically exhibit Lewis acidity exhibited by metal cations, leading to DOL ring opening polymerization. Moreover, initiators have a significant impact on the physicochemical and electrochemical properties of the electrolyte, including the DOL conversion rate, PDOL molecular weight, electrolyte state, ion conductivity, and oxidation potential. Therefore, an in-depth exploration of the role of initiators is essential for developing PDOL solid electrolytes with desirable application properties.

**Table 1:** Classification of different initiators and performance comparison of the PDOL electrolyte initiated by them

Type of initiator	Initiator	Initiation factor	Initiator content	DOL conversion rate (%)	PDOL molecular weight	Electrolyte state	Ionic conductivity (mS cm^−1^)	Oxidation potential (V)	Refs
Lithium salt	LiPF_6_	Protonic acid	2.0 M	91	~ 53 000 (Mn)	Gel	3.8	4.6	[58]
	LiPF_6_	Protonic acid	1.0 wt%	98.5	23 588 (Mw)	Solid	0.28	5.2	[68]
	LiDFOB	Protonic acid	0.3 M	90	/	Gel	0.39	5.1	[75]
	LiBF_4_	Protonic acid	0.2 M	81.2	–	Solid	0.3	4.9	[76]
	LiFSI	Protonic acid	3.5 M	75	–	Gel	7.9	4.7	[77]
Aluminum salt	Al(OTf)_3_	Lewis acid	0.5 mM	86	15 000 (Mn)	Solid	1.1	5.0	[64]
	Al(OTf)_3_ + AlF_3_	Lewis acid	0.5 mM + 0.3 M	12	18 000 (Mw)	Solid	1.8	4.7	[66]
	AlI_3_	Lewis acid	600 ppm	84	–	Gel	–	–	[57]
Tin salt	Sn(OTf)_2_	Lewis acid	2.0 mM	90	12 417 (Mw)	Gel	6.2 × 10^–2^	4.9	[78]
	SnF_2_	Lewis acid	1.5 wt%	92	29 552 (Mw)	Solid	1.7 × 10^–2^	5.0	[79]
Other salts	ZnCl_2_	Lewis acid	25 wt%	–	51 798 (Mn)	–	–	–	[50]
	Sc(OTf)_3_	Lewis acid	1.0 mM	84	48 963 (Mn)	Solid	8.7 × 10^–2^	~ 5.0	[80]
	Mg(OTf)_2_	Lewis acid	1.0 wt%	79.9	~ 57 000 (Mn)	Solid	0.5	4.3	[81]
Acidized materials	Acidized Al_2_O_3_	Protonic acid	4.0 wt%	89.6	32 000 (Mn)	Gel	3.37	4.5	[82]
	Acidized CNT paper	Protonic acid	–	–	–	Gel	–	–	[56]
Organic materials	TB	Protonic acid	3.0 wt%	89	–	Solid	1.16	4.8	[83]
	S(C_2_H_4_O_4_)	Lewis acid	0.3 M	88.2	5 391 (Mn)	Solid	0.38	4.7	[84]
	HFiP	Nucleophilic	0.5 wt%	67	–	Gel	1.6	4.7	[85]

#### Polymerization Initiated by Lithium Salt

**Fig. 5:**
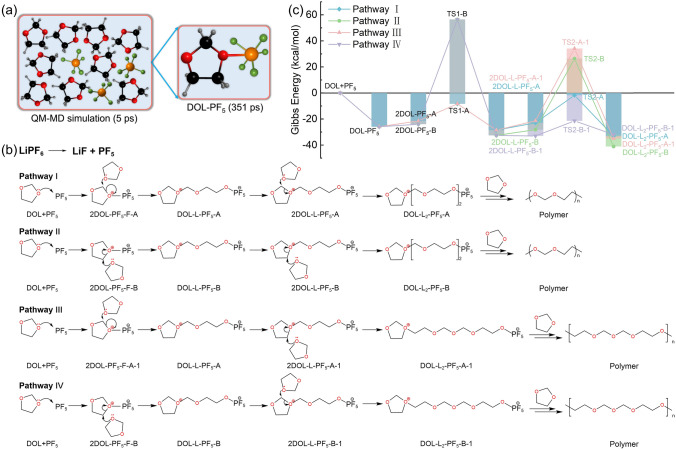
Schematic diagram of LiPF_6_-initiated DOL polymerization process. **a** Structure of DOL-PF_5_ obtained from the 3ps QM-MD simulation of PF_5_-DOL system. **b** Four possible paths of DOL ring-opening polymerization under the action of PF_5_ and **c** reaction Gibbs free energy [86]

In the pertinent research on the polymerization of DOL to form PDOL electrolyte, the predominant use of LiPF_6_ as an initiator is attributed to its high initiation efficiency and excellent cycling performance. Hence, a more comprehensive comprehension of the process of LiPF_6_-initiated DOL polymerization can enable the derivation of improved DOL polymerization techniques. Tao Cheng [86] used DFT and quantum mechanics (QM) molecular dynamics (MD) simulation to calculate the most possible reaction path for LiPF_6_-initiated DOL ring-opening polymerization. The QM-MD simulation results, as shown in Fig. 5a, indicate that PF_6_^−^ decomposes into F^−^ and leaves behind PF_5_ with free radical properties, while DOL forms a P–O bond with PF_5_ after 351 fs, and other DOL molecules approach the DOL-PF_5_ system, which is the initial state of DOL ring-opening reaction. The authors proposed a possible polymerization path, where LiPF_6_ first decomposes into LiF and PF_5_ free radicals, and the lone pair electrons of the O atom in DOL combine with PF_5_ to form DOL-PF_5_, where the C–O bond in DOL-PF_5_ is activated and other approaching DOL molecules attack the C atom to form DOL-L-LF_5_ via ring-opening reaction. The C–O bond in unopened DOL-L-LF_5_ is further activated, achieving continuous polymerization. In the above polymerization simulation, there are two types of activated C–O bonds attacked by free DOL: –O–C–O– and –O–C–C–O–, denoted as A and B, respectively. Four more detailed possible polymerization paths can be formed by alternating ring-opening of DOL at the two different C–O bond positions of A and B during the ring-opening process (Fig. 5b). The authors calculated the free energy of the four polymerization reaction pathways and found that the energy barrier of Pathway I that needs to overcome is the lowest (Fig. 5c), making it the most reasonable polymerization path for DOL.

**Fig. 6:**
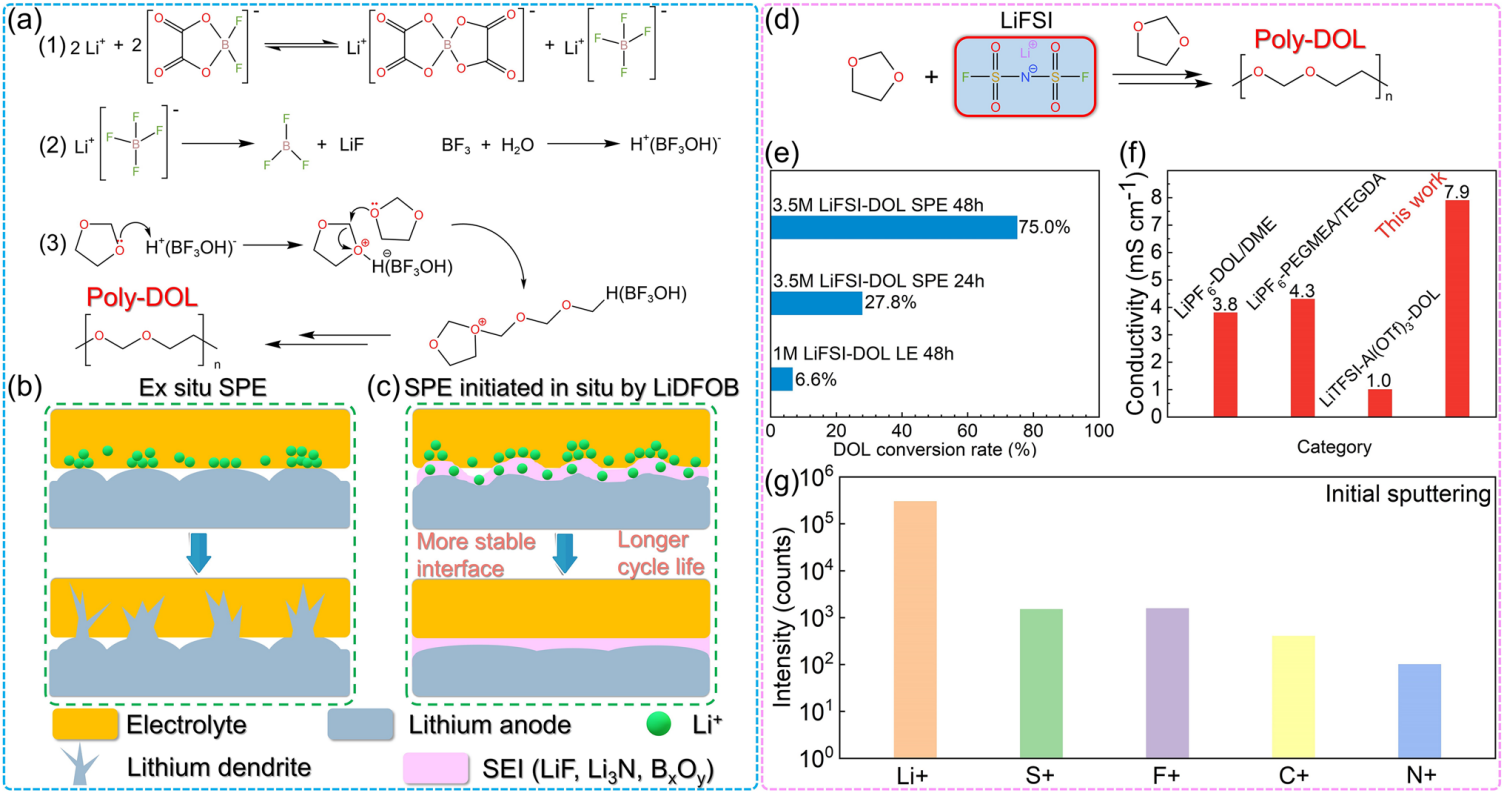
LiDFOB (LiBF_4_) or LiFSI initiated DOL polymerization. **a** Schematic illustration of the LiDFOB initiated DOL ring-opening polymerization process. **b** Interphase evolution of ordinary non-in situ polymer electrolytes leads to incomplete interface contact, resulting in uneven ion transport and rapid growth of lithium dendrites [87]. **c** LiDFOB in-situ initiated polymerization of PDOL electrolyte generates SEI rich in boron oxide compounds at the electrolyte/lithium anode interface, which guides ion uniform deposition, resulting in a more stable interphase and longer cycle life. **d** LiFSI initiates DOL ring-opening polymerization process. **e** DOL polymerization conversion rate at different LiFSI content and standing time. **f** Comparison of ionic conductivity of PDOL gel electrolyte initiated by LiFSI with other PDOL electrolytes. **g** TOF–SIMS of C + , F + , S + , N + and Li + species on the lithium surface after cycling in the gel electrolyte: species concentration at initial sputtering [77]

Apart from LiPF_6_, other lithium salts are also widely used as DOL ring-opening initiators due to the trace amounts of protonic acids they generate. Li Yang [87] investigated the mechanism of lithium difluorooxalatoborate (LiDFOB) as a ring-opening initiator. Since LiDFOB often exists partially as LiBF_4_, its initiation mechanism is analogous to that of LiBF_4_ (Fig. 6a1). LiBF_4_ has the ability to generate BF_3_, and BF_3_ reacts with H_2_O to generate H^+^(BF_3_OH)^−^ (Fig. 6a2), which function as potent protonic acids. H^+^ on H^+^(BF_3_OH)^−^ reacts with the oxygen atom on the DOL ring, initiating the ring-opening polymerization of DOL and generating long chains of PDOL (Fig. 6a3). The Li//Li symmetric battery assembled with LiDFOB initiated polymer electrolyte was tested, which has much longer cycle life than the symmetric battery with ex-situ polymerization. In ex-situ polymerized batteries, there are a large number of defects in the interphase between the electrolyte and the anode, and ions will aggregate at the contact point, accelerating the growth of dendrites (Fig. 6b). In LiDFOB in-situ initiated PDOL batteries, SEI is composed of LiF, Li_3_N, and B_*x*_O_*y*_ derived from initiators, which protect the lithium metal anode while guiding ion uniform deposition, ultimately achieving a more stable interface and longer cycle life (Fig. 6c).

Further, a scheme for initiating DOL ring-opening with high concentration of lithium difluorosulfonamide (LiFSI) has been proposed. The authors were able to initiate the DOL ring-opening reaction with only higher concentration of LiFSI without adding additional lithium salts (Fig. 6d) [77, 88, 89]. With a concentration of 3.5 M LiFSI, the DOL polymerization conversion rate can achieve 75% (Fig. 6e), and the resulting gel electrolyte demonstrates an impressive ionic conductivity of 7.9 mS cm^−1^ (Fig. 6f). The elevated concentration of lithium salt leads to the dissociation of higher concentrations of Li^+^, thereby playing a crucial role in enhancing Li^+^ diffusion capability. Time of flight secondary ion mass spectrometry (TOF–SIMS) analysis of the lithium surface SEI cycled in the gel electrolyte shows that the N, F, and S elements from LiFSI gradually decrease as the sputtering time increases, indicating the formation of a gradient structure SEI (Fig. 6g). It shows that the lithium surface is covered with a LiF-rich SEI layer, which can effectively protect the lithium metal anode.

#### Polymerization Initiated by Aluminum Salt

**Fig. 7:**
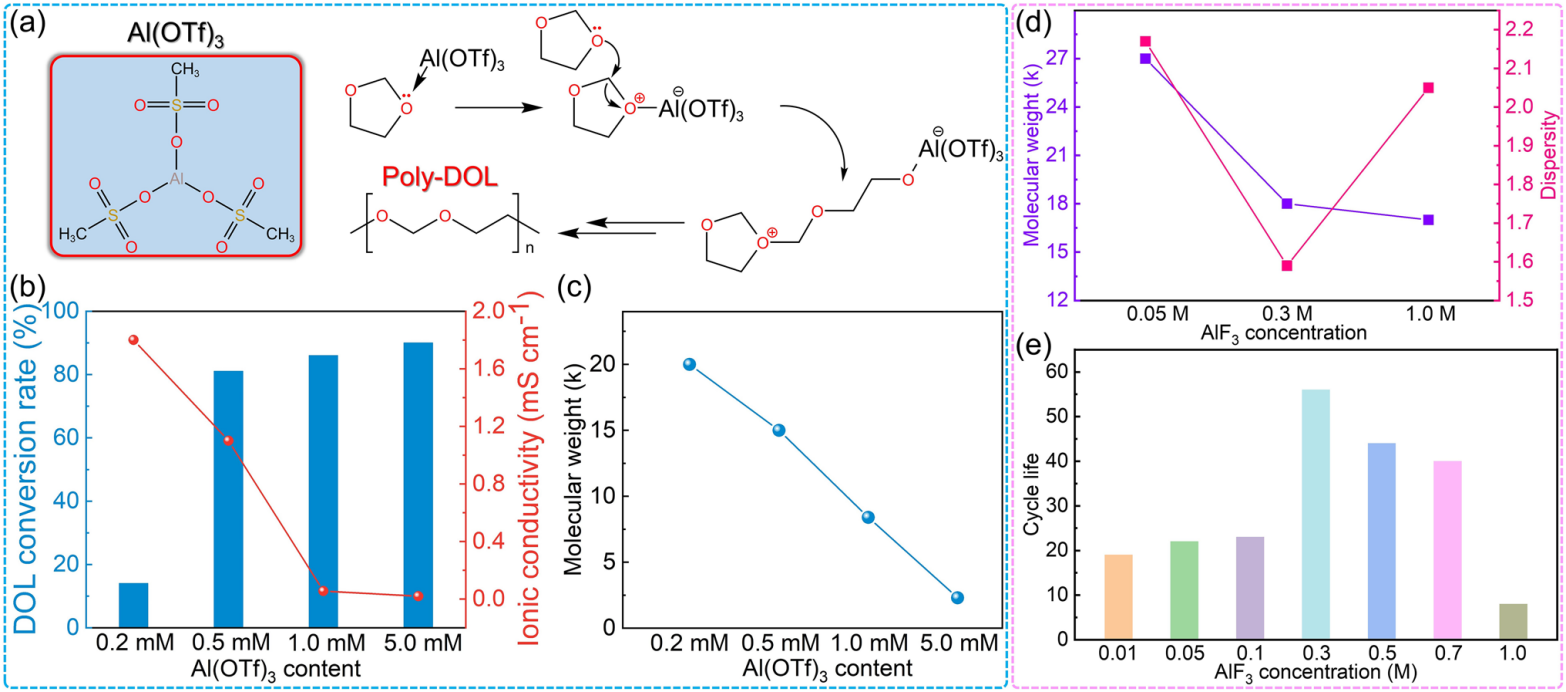
Aluminum salt initiates DOL polymerization. **a** Mechanism of DOL polymerization initiated by Al(OTf)_3_. **b** DOL polymerization conversion rate and **c** molecular weight of PDOL initiated by different aluminum salt initiators content [64]. After adding different contents of AlF_3_, **d** the molecular weight and dispersity of PDOL, and **e** cycle life of PDOL [66]

Cations in metal salts generally exhibit Lewis acidity and are used as DOL ring-opening initiators [90]. Lynden A. Archer [64] used trace amounts of aluminum trifluoromethanesulfonate (Al(OTf)_3_) to initiate in-situ polymerization of DOL. The aluminum cation in Al(OTf)_3_ exhibits a strong electron-attracting ability and thus preferentially coordinates with the lone pair of electrons on the oxygen atom of the DOL ring. This coordination interaction initiates the ring-opening polymerization of DOL, which subsequently proceeds via a chain-growth mechanism (Fig. 7a). Aluminum salt initiation does not require the involvement of additional trace amounts of water as lithium salt initiation does, and its lower usage indicates its high initiation efficiency. The authors compared the effects of different aluminum salt initiators on the properties of PDOL electrolyte and found that at an initiator concentration of 0.5 mM Al(OTf)_3_, it simultaneously achieved a polymerization conversion rate up to 81% for DOL and a high ionic conductivity of 1.1 mS cm^−2^ (Fig. 7b). Moreover, the authors discovered that as the concentration of aluminum salt increases, the molecular weight of the polymer decreases due to the rise in initiation sites, leading to a decrease in chain length (Fig. 7c). The in-situ polymerized solid-state LiFePO_4_ (LFP)//Li batteries maintain high and stable coulombic efficiency during ultra-long cycles.

Building upon this work, the Lynden A. Archer group [66] incorporated AlF_3_ as a co-initiator with the original Al(OTf)_3_ to enable application with high-voltage cathode. The addition of AlF_3_ has a similar effect as the initiator. As its content increases, the molecular weight of PDOL decreases. At a concentration of 0.3 M, the weight average molecular weight is 18 kDa and the dispersion is 1.59 (Fig. 7d). The AlF_3_ enables the application of PDOL on high-voltage LiNi_0.6_Co_0.2_Mn_0.2_O_2_ (NCM622) layered cathode materials. When 0.3 M AlF_3_ is added, its stable cycle times can reach up to nearly 60 times (Fig. 7e), and the charge/discharge voltage curves also show good cycling stability.

#### Polymerization Initiated by Other Typical Initiators

Simultaneously serving as a functional additive, initiator is now being used in PDOL electrolytes. Weidong Zhou [79] employed SnF_2_ as a superior interfacial stabilizer to initiate ring-opening polymerization of DOL. SnF_2_ can react in situ to generate interphase protective species Li_*x*_Sn during battery operation, which can effectively prolong the working life of lithium-ion batteries.

**Fig. 8:**
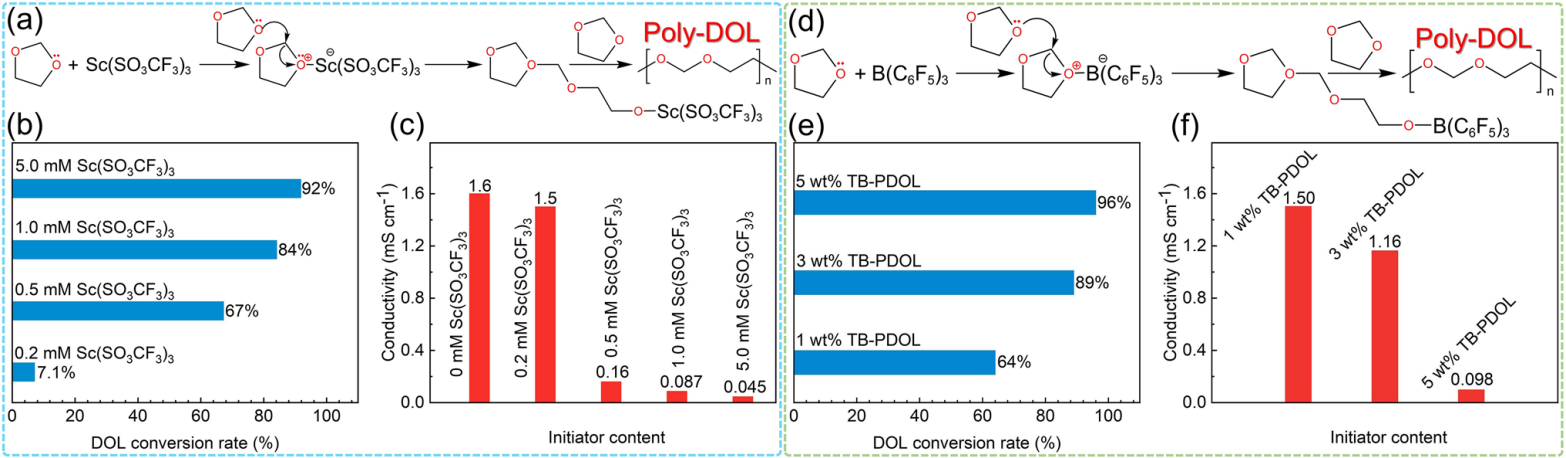
Scandium salt and TB initiate DOL polymerization. **a** Mechanism of DOL polymerization initiated by Sc(SO_3_CF_3_)_3_. **b** DOL polymerization conversion and **c** ionic conductivity of PDOL electrolyte initiated with different initiator concentrations [92]. **d** TB initiates the DOL polymerization process. **e** DOL polymerization conversion rate and **f** room temperature ionic conductivity initiated by different TB contents [83]

Transition metal salts are also used as alternative initiators for DOL polymerization [91]. Shi Wang [92] employed minute quantities of Sc(SO_3_CF_3_)_3_ to in-situ polymerize and fabricate PDOL solid electrolytes. Similar to traditional aluminum salts, Lewis acidic Sc^3+^ opens the DOL ring and initiates polymerization (Fig. 8a). Comparative experiments showed that with the addition of 1.0 mM Sc(SO_3_CF_3_)_3_, the polymerization conversion rate of DOL reaches 84% (Fig. 8b) and the ion conductivity reaches 8.7 × 10^–5^ S cm^−1^ (Fig. 8c). This trace initiator exhibits high initiation efficiency and good polymerization results.

Except for most salt initiators, organic initiators such as tris(pentafluorophenyl) borane (TB) have also been reported [83]. Its initiation mechanism is also inclined toward salt initiation, and B atom with electron attraction ability will attack the lone electron pairs of oxygen atoms on the DOL ring, leading to the ring-opening of the DOL (Fig. 8d). Incorporation of 3 wt% TB initiator resulted in the attainment of 89% polymerization conversion of DOL (Fig. 8e), while retaining a high room temperature ionic conductivity of 1.16 mS cm^−1^ (Fig. 8f). Surface XPS analysis of the lithium sheet after cycling reveals the formation of a substantial quantity of LiF at the interface, a critical factor in enhancing interfacial stability. Besides, the O–B–O bond present in B_*x*_O_*y*_ produced by TB decomposition facilitates the creation of cross-linked covalent frameworks, which can culminate in the formation of a more robust SEI.

### Nucleophilic Polymerization of DOL

**Fig. 9:**
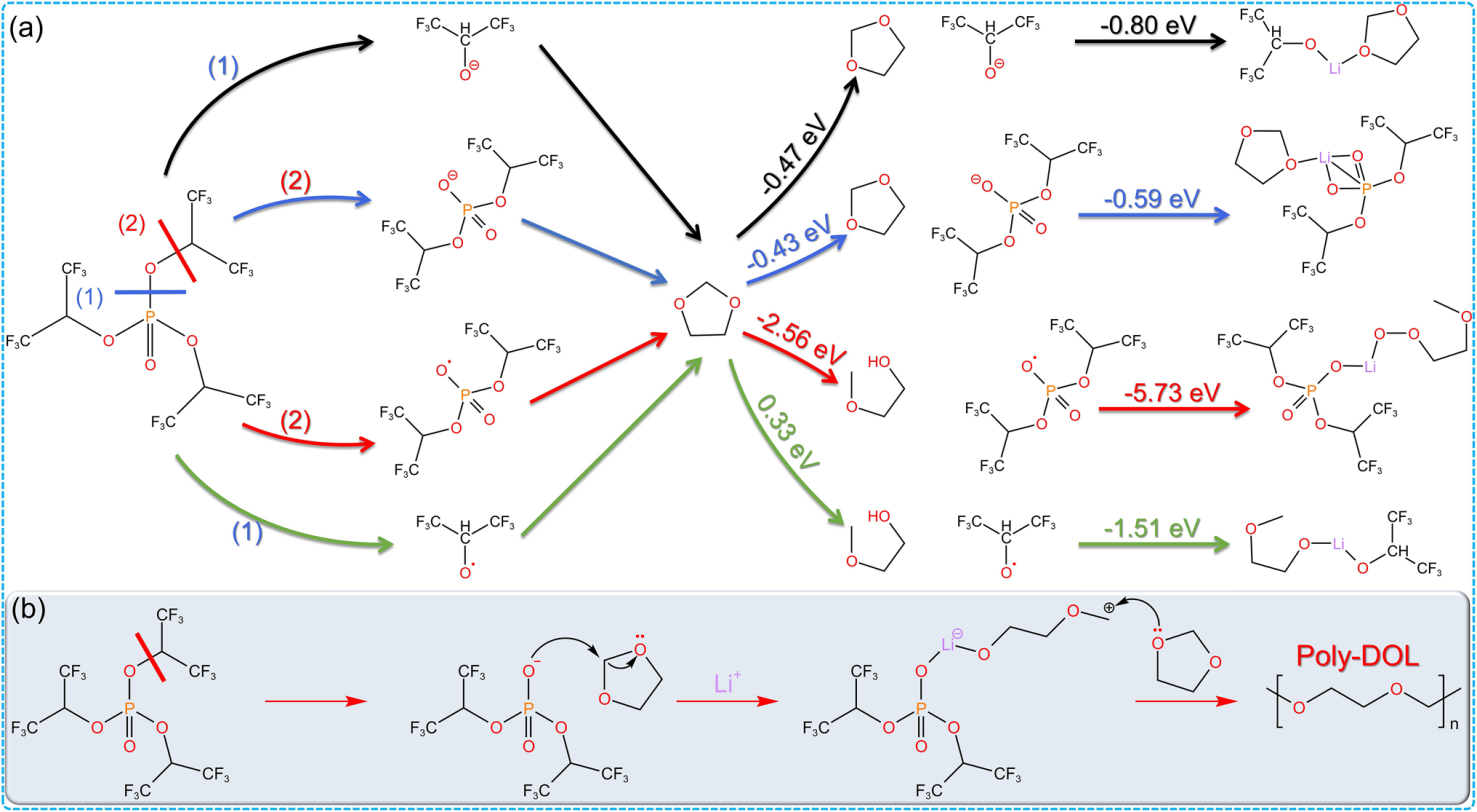
Nucleophilic polymerization mechanism of DOL. **a** Some free radicals generated from the decomposition of HFiP and their free energy for ring-opening reactions with DOL. **b** Schematic diagram of HFiP initiated nucleophilic polymerization of DOL [85]

In the second stage of electrophile-initiated DOL ring-opening polymerization, it also falls under nucleophile initiation: the oxygen atom with a lone pair of electrons on the free DOL ring attacks the carbon atom in the –O–C–O– moiety of the initiator-bound DOL ring, promoting the chain reaction of DOL polymerization. Some initiators can also undergo nucleophilic initiation with the assistance of Li^+^ in the first stage. Shuting Yang [85] used tris(1,1,1,3,3,3-hexafluoroisopropyl) (HFiP) in situ to initiate DOL ring-opening polymerization by nucleophilic pattern into gel cells. When HFiP is in solution, the P–O and C–O bonds are easily broken [93, 94], and the charged groups after cleavage will attack the oxygen atoms on the DOL ring, leading to DOL ring-opening. DFT calculations show that the ring-opening process under the initiation of cations containing P–O bonds releases 2.56 eV of energy (red line path), which is the most possible initiation mechanism (Fig. 9a). In this pathway, the breaking of the C–O bond generates a negatively charged oxygen atom. This nucleophilic reagent attacks the carbon atom in the –O–C–O– moiety of the DOL ring, opening the ring and initiating the polymerization reaction of DOL, facilitated by the simultaneous action of the electrophilic Li^+^ (Fig. 9b). HFiP effectively initiates nucleophilic polymerization of DOL, and its decomposition products have no any impact on the battery, demonstrating excellent stability.

## Interface of PDOL Electrolyte

**Fig. 10:**
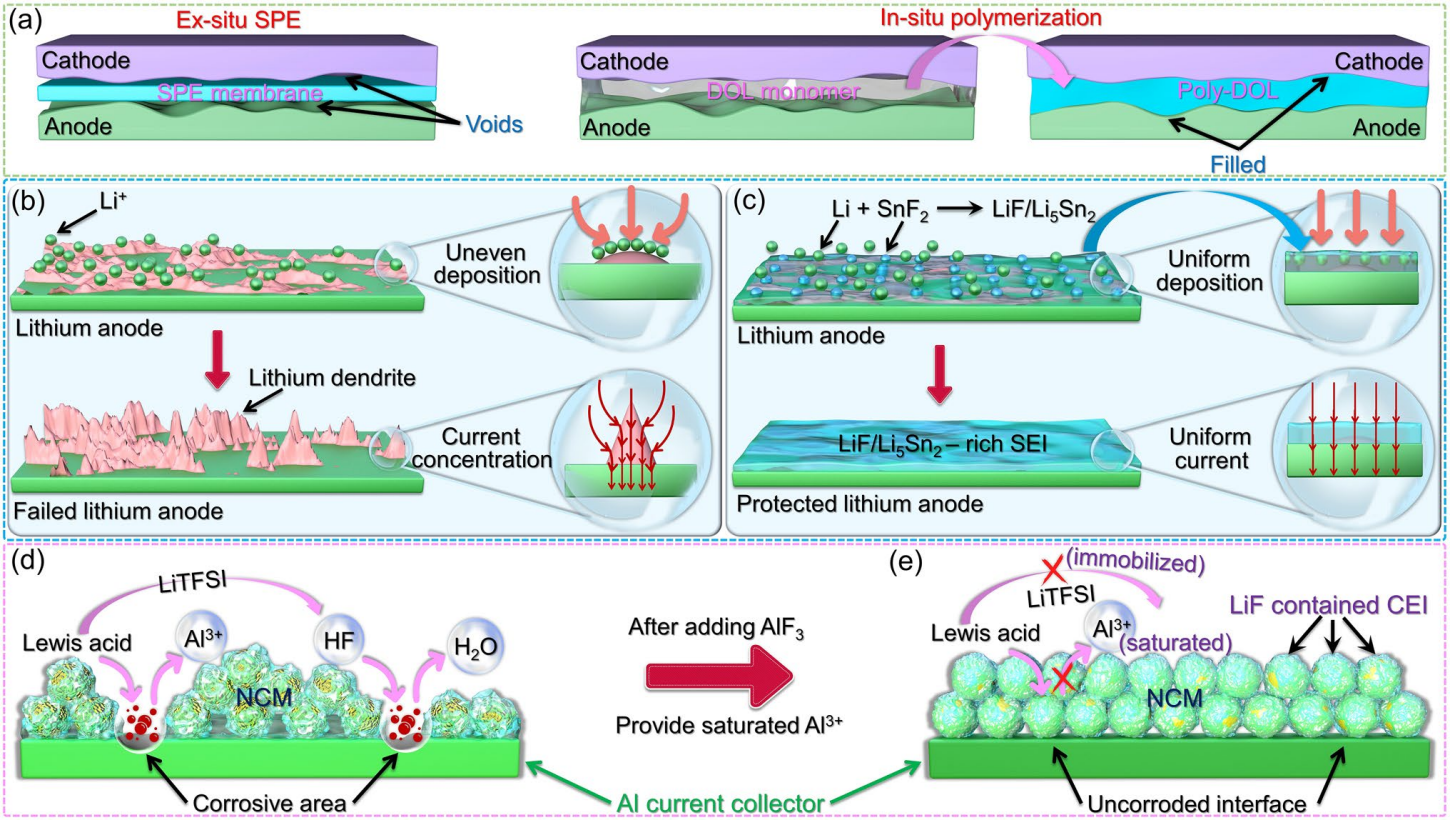
Contact mode and interface optimization between PDOL electrolyte and solid-state electrode. **a** Differences in interface contact modes between electrolytes and solid-state electrodes in ex-situ polymerization and in-situ polymerization [64]. **b** The uneven deposition of lithium ions on common lithium metal anodes leads to rapid growth of lithium dendrites: in areas where lithium ions are concentrated and deposited, it will further guide the aggregation of ions and currents, leading to the growth of dendrites. **c** In the SnF_2_-initiated PDOL electrolyte, the initiator undergoes a reaction with lithium to generate Li-Sn alloy, and it then forms a robust LiF/Li_5_Sn_2_-rich SEI incorporating LiF. The SEI facilitates the homogeneous deposition of lithium ions and the even distribution of current, providing protection to the lithium metal anode [79]. **d** Mechanism diagram of proton corrosion of aluminum current collector caused by Lewis acid interface reaction. **e** Mechanism diagram of aluminum current collector corrosion inhibition after adding AlF_3_ [66]

The interface engineering between electrolyte and electrode will have a serious impact on the electrochemical performance and operational life of SSLB. Traditional solid-state electrolytes, which have been extensively studied, are often prepared in an ex-situ manner. However, these solid-state electrolytes fail to achieve the wetting effects observed in liquid electrolytes, leading to the formation of significant voids at the interface between the electrolyte and electrode. This phenomenon hampers Li^+^ diffusion and contributes to localized current concentration. In contrast, PDOL-based electrolytes can be polymerized from liquid DOL monomers. As a result, introducing DOL monomers and initiators into the battery will enable automatic wetting of the electrode interface. The in-situ liquid–solid transition characteristic of PDOL retains this adaptability, allowing the solid PDOL to effectively fill the interface pores between the electrolyte and electrode, ensuring the continuity of Li^+^ diffusion (Fig. 10a).

The energy density advantage of SSLB is related to the lithium metal anode, and the active lithium metal is prone to various side reactions and failure. Constructing SEI protection for lithium metal is an effective method to extend the electrode service life [95–101]. SEI requires high electrochemical stability and ion transport capability, and the reported SEI components mainly include fluorides [95, 102–107] (LiF), nitrides [87, 108] (Li_3_N), carbides [107, 109–111] (Li_2_CO_3_, LiC_*x*_), alloys [79, 112–116] (Li–Cu, Li–Sn, Li–Se), etc. Components such as LiF and Li_3_N are mainly derived from the decomposition of lithium salts containing F and N elements, while the formation of carbides is often related to graphite anodes or contact with CO_2_ in the air. The alloy composition is mainly related to composite anodes or metal salt additives. To elucidate the protective mechanism of SEI on lithium metal, we employ Li–Sn alloy as an exemplary case. Weidong Zhou [79] pioneered the utilization of SnF_2_ initiators to initiate DOL polymerization for the fabrication of solid-state batteries. CV testing was conducted on a Li//Fe battery assembled with PDOL initiated by SnF_2_. A small lithiation current was observed at 0.35 and 0.62 V, which could be attributed to the formation of Li_*x*_Sn. The X-ray photoelectron spectroscopy (XPS) of F and Sn elements on the surface of the freshly synthesized PDOL electrolyte membrane and the recycled one from Li//Li battery after cycles show that SnF_2_ forms a composite SEI enriched with LiF and Li_*x*_Sn. Finite element simulation (FES) is utilized to depict the potential field and Li^+^ flux distribution at the interface between the electrolyte and lithium metal. In conventional electrolytes, following the initial non-uniform deposition of lithium ions on the surface of the lithium anode, subsequent Li^+^ flux is also unevenly distributed, leading to the accelerated growth of lithium dendrites (Fig. 10b). However, the interface electric field of the composite SEI containing Li_5_Sn_2_/LiF is relatively smoother, resulting in a slower voltage decrease. Due to the significantly higher Li^+^ diffusion coefficient of Li_5_Sn_2_ than that of LiF, Li^+^ flux primarily distributes on Li_5_Sn_2_, which has superior ionic conductivity, while passing through the SEI layer. This facilitates the uniform distribution of Li^+^ flux and suppresses the formation of lithium dendrites (Fig. 10c).

**Fig. 11:**
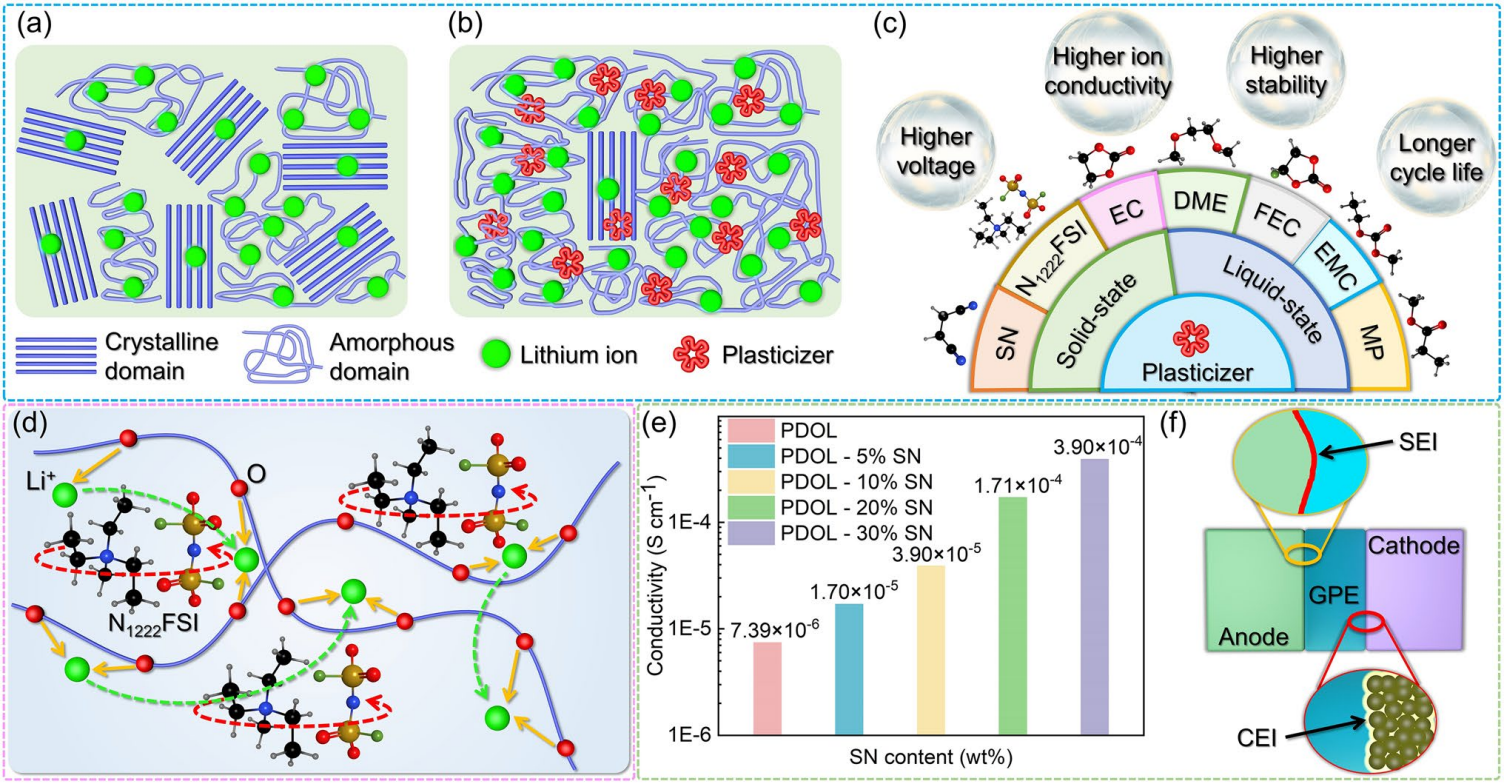
Improvement of PDOL electrolyte performance by plasticizers. **a** PDOL polymers without plasticizers possess a significant amount of crystalline domains in their structure, which hinders the mobility of polymer chains. **b** The addition of plasticizers to PDOL increases the amorphous domains, facilitating chain segment mobility and resulting in enhanced ion conductivity. **c** The reported types of plasticizers and their enhancements on the performance of PDOL polymers. **d** The internal rotational motion of N_1222_FSI leads to orientation disorder and the generation of lattice vacancies in PDOL, thereby enhancing ion conductivity [87]. **e** The effect of different SN contents on the ion conductivity of PDOL GPE. **f** The CEI and SEI gradually formed during the cycling process of GPE protect the cathode and anode, respectively [75]

The lack of satisfactory high voltage tolerance in DOL is attributed to its instability toward highly charged cathodes, which leads to unwanted reactions. Since DOL monomers inevitably exist in PDOL electrolytes, preventing direct contact between PDOL electrolytes and cathode materials will effectively mitigate undesired side reactions. Coating the cathode [117–119] or constructing a cathode electrolyte interphase (CEI) has been identified as an effective solution. Lynden A. Archer [66] achieved the protection of high-voltage NCM cathodes in PDOL by incorporating AlF_3_, and the author also explained the reason why AlF_3_ enhances the high-voltage performance. The Lewis acid in the electrolyte generates protons at the interface, which react with the Al_2_O_3_ on the surface of the aluminum current collector to produce soluble Al^3+^ and react with lithium salt to generate HF. Both reactions can corrode the aluminum current collector (Fig. 10d). The addition of AlF_3_ forms a saturated Al^3+^ solution, which prevents the decomposition of Al_2_O_3_ and immobilizes TFSI^−^, thereby preventing corrosion of the aluminum current collector (Fig. 10e). Furthermore, the addition of succinonitrile (SN) has been demonstrated to form a thin and stable CEI, which helps maintain the stability of the cathode/PDOL interface and effectively prolong the battery’s operational lifespan (Fig. 11f) [75].

## Composites of PDOL Electrolyte

In the context where SSLB have not yet achieved large-scale commercialization, it is evident that conventional SPE face several unresolved problems. One of the most significant modification strategies for traditional SPE is the addition of additives, which is preparing composite electrolytes. The types of additives mainly include polymer fillers, inorganic fillers, and support scaffolds, among others. The general function of additives is to disrupt the arrangement of polymer segments, reduce the glass transition temperature of polymer, and thereby enhance its room temperature ionic conductivity. The addition of polymer fillers often enhances the toughness of the polymer to improve the interface contact problem. Inorganic fillers are the most commonly used additives to improve the electrochemical performance of polymers. They have significant effects in increasing the polymer’s ionic conductivity, widening its electrochemical stability window, and enhancing its thermal stability. The addition of inorganic SiO_2_ to pentaerythritol tetraacrylate (PETEA) can significantly enhance its room temperature ionic conductivity, increasing it by an order of magnitude to reach 1.93 × 10^–3^ S cm^−1^ [104]. When MoSe_2_ is added to PVDF, the high-voltage tolerance of the SE is increased from 4.3 to 4.7 V [116]. This improvement in voltage stability can enhance the performance and safety of the electrolyte in high-voltage applications. Polymer electrolytes often lack the compressive mechanical strength to resist tearing caused by external pressure. Introducing a support skeleton can effectively address this issue. The support skeleton provides structural integrity and enhances the mechanical properties of the polymer electrolyte, allowing it to withstand external forces and reduce the risk of tearing or failure. The relatively weak mechanical strength of poly (propylene carbonate) (PPC) prevents it from achieving self-support. However, by incorporating a cellulose support skeleton, the tensile strength of PPC can be enhanced to reach 15 N mm^−2^, thereby satisfying the application requirements of electrolytes [120]. Conventional SPE has achieved various performance improvements through the addition of additives, which also offer research directions for future SPE modifications.

Despite demonstrating exceptional performance in lithium-ion batteries, PDOL falls short in fully satisfying the practical requirements for its application in terms of volume stability, flexibility, ion conductivity, electrochemical window, high voltage stability, mechanical strength, and thermal stability. Similar to the modification scheme of conventional SPE, combing with other advantageous materials is an effective method to improve its electrochemical properties. Researchers have explored the incorporation of plasticizers, support skeletons, and inorganic fillers to achieve substantial enhancements across various performance parameters. These findings indicate a promising development trajectory for the practical utilization of PDOL in the future.

### Plasticizers

Although the in-situ polymerization approach can significantly enhance the interface contact between the electrolyte and electrode, the polymerization of DOL alone may not be sufficient to achieve optimal results for practical applications. Mainly attributed to two aspects: Firstly, PDOL polymers exhibit partial crystallization, resulting in poor plasticity, which cannot adapt to the defects caused by the repeated volume changes of electrode materials during cycling. Plasticizers can significantly enhance the plastic deformation ability of PDOL electrolytes, actively adapt to changes in electrode volume, and reduce defects. Secondly, the high degree of polymerization of PDOL and its ionic conductivity do not satisfy the requirements for high energy density batteries. The large number of crystalline domains inside PDOL hinder the peristalsis of the chain segments, and the migration of ions cannot meet the application requirements (Fig. 11a). The introduction of plasticizers increases the amorphous domains of PDOL polymers, leading to a significant enhancement in ion conductivity (Fig. 11b).

The reported plasticizers for PDOL electrolytes encompass two categories: solid-state and liquid-state, both of which primarily consist of small molecule monomers. Solid plasticizers will retain more of the advantages of PDOL polymers as solid electrolytes, while liquid plasticizers exhibit excellent performance in improving the ionic conductivity of PDOL polymers. Higher electrode stability and higher ion conductivity are the most prominent contributions of plasticizers to PDOL polymers. Furthermore, certain plasticizers have demonstrated the ability to enhance high voltage tolerance and prolong the cycle life of PDOL polymers, further optimizing their overall performance (Fig. 11c). Plasticizers are simple and extremely effective in improving the performance of PDOL polymers.

Li Yang [87] incorporated the organic ionic plastic crystal triethylmethylammonium bis (fluorosulfonyl) imide (N_1222_FSI) into the PDOL electrolyte and evaluated the impact of various plasticizers on the performance of PDOL. As the N_1222_FSI content increased, the researchers observed a corresponding rise in ion conductivity and a decrease in the degree of polymerization of the electrolyte. The researchers posit that the addition of N_1222_FSI to the PDOL electrolyte provides two pathways for Li^+^ transport. One is facilitated by the coordination/dissociation of N_1222_FSI with the oxygen atoms in PDOL, while the other is enabled by the orientation disorder and lattice vacancies generated by ion rotation inside N_1222_FSI (Fig. 11d). These mechanisms are considered to be the primary factors contributing to the observed improvement in the ion conductivity of the polymer when compared to pure PDOL electrolyte. Furthermore, at additive levels between 0 and 40 wt%, the electrochemical stability window of the PDOL electrolyte surpasses 5.8 V, thereby providing a solid foundation for practical applications.

1,2-dimethoxyethane (DME) has been extensively used as a plasticizer for PDOL electrolytes and has shown favorable balance performance [58, 59, 63, 67, 77, 85, 121]. Maoxiang Jing [122] investigated in detail the effect of DME addition on PDOL polymerization conversion and found that with increasing DME content, the polymerization conversion of DOL decreased. The Arrhenius equation was employed by the author to fit and compute the activation energy at varying ratios, based on the ion conductivity measurements at different temperatures. The analysis revealed that the addition of DME to PDOL electrolyte reduces the lowest activation energy to as low as 0.1 eV, providing greater facilitation of Li^+^ diffusion across a broader temperature range. However, the introduction of a higher concentration of small molecule DME into PDOL-based polymer electrolytes has been shown to decrease the electrochemical stability window. Therefore, careful regulation of the DOL and DME ratio is essential to achieve a balance between ion conductivity and electrochemical stability window.

The plastic crystalline plasticizer, SN, has been found to enhance the ion conductivity of PDOL polymer [75]. It was observed that the ion conductivity of PDOL steadily increased as the SN content increased (Fig. 11e). The ion conductivity measurements conducted at varying temperatures revealed that the addition of SN resulted in a lower ion migration barrier and higher ion conductivity. Nevertheless, akin to small molecule plasticizers like DME, the incorporation of SN into PDOL was found to decrease the electrochemical stability window. The author balanced the performance of both and chose to add 30 wt% SN to achieve better comprehensive performance (Fig. 11e). Furthermore, the authors discovered that the cathode in PDOL with the addition of SN forms a thin and stable CEI after cycling (Fig. 11f). This interphase reduced the Li^+^ diffusion barrier and effectively suppressed interface side reactions. Several studies have further added fluoroethylene carbonate (FEC) [81, 123–125] on the basis of adding SN, and have observed further improvements in both the ionic conductivity and electrochemical stability window of the polymer.

In the traditional liquid electrolyte system, a combination of ethylene carbonate (EC) and ethyl methyl carbonate (EMC) is used to dissolve the DOL ring-opening initiator Sc(OTf)_3_ and employed as plasticizers for PDOL polymer [126, 127]. Pre-dissolved initiators can initiate DOL polymerization more uniformly to prevent local polymerization. The plasticizing properties of EC/EMC can also significantly enhance the ion conductivity of the polymer. In addition, using the low-melting plasticizer methyl propionate (MP) [128] and adjusting the ratio between DOL, MP, and LiTFSI can optimize the ion conductivity, with the highest room-temperature ion conductivity exceeding 2.9 mS cm^−1^. The PDOL-MP composite electrolyte demonstrates suitability for the assembly of high-voltage NCM811//Li batteries, enabling the attainment of a relatively stable cycling performance.

### Support Skeleton

**Fig. 12:**
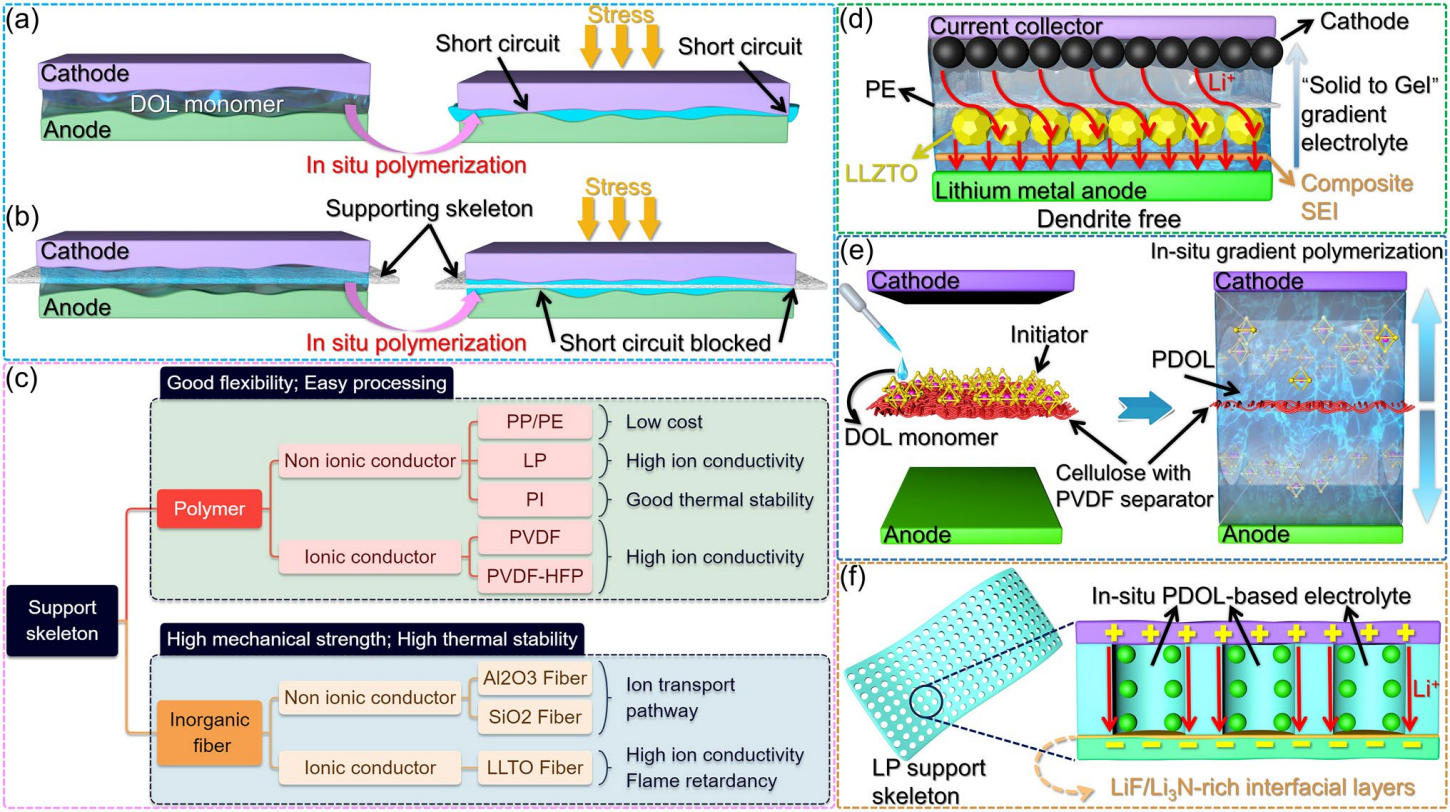
Function and classification of support skeleton. **a** In the absence of a support skeleton, the electrode may come into contact and experience short circuits under external pressure during the in-situ polymerization process of DOL. **b** The presence of support skeletons effectively serves as a mechanical barrier, preventing electrode contact and mitigating the risk of short circuits during the in-situ polymerization process. **c** The types of support skeletons and their corresponding advantages, they can be broadly categorized into two main groups: polymer-based and inorganic-based materials. **d** Schematic diagram of the battery structure after DOL gradient polymerization initiated by modified PE, the densely polymerized PDOL solid electrolyte adjacent to the anode serves as a protective barrier for the lithium metal, while the partially polymerized PDOL gel electrolyte in close proximity to the porous cathode effectively permeates and infiltrates the electrode [131]. **e** Schematic diagram of bidirectional gradient polymerization of DOL on modified cellulose paper [132]. **f** Schematic diagram of the interphase layer between electrolyte and LP to enhance ion conductivity [76]

The high ionic conductivity exhibited by the PDOL polymer at room temperature is attributed to its comparatively lower glass transition temperature, which correlates with its reduced mechanical strength under the same conditions. As a consequence of its low mechanical strength, the aforementioned electrolyte is inadequate for effectively separating the cathode and anode, thereby compromising the battery’s stability under external pressure and exacerbating the issue of dendrite growth of the lithium metal anode, all of which are indispensable factors for the optimal performance of a lithium-ion battery (Fig. 12a). Consequently, it is imperative to reinforce the PDOL polymer with a robust support skeleton, capable of maintaining electrode isolation even in the face of external pressure or deformation. This measure is vital for sustaining normal operation under challenging environmental conditions (Fig. 12b). The support skeletons mentioned in literatures primarily encompass polymer and inorganic materials, each boasting distinct advantages. Polymer skeletons are renowned for their superior flexibility and ease of processing, while inorganic skeletons exhibit higher mechanical strength and ion conductivity. These materials can be further classified into ionic conductors and non-ionic conductors based on their capacity to conduct Li^+^ ions. Within their compositions, these materials possess unique functionalities that cater to a diverse array of requirements (Fig. 12c). The performance of the battery varies depending on the choice of the support material, and thus, identifying a functionally balanced support skeleton is a crucial research avenue for achieving high-performance PDOL polymer solid electrolytes.

#### Polymer Support Skeleton

Polyethylene (PE) and polypropylene (PP) separators are commonly employed as support skeletons for traditional liquid batteries thanks to their stable electrochemical properties and good mechanical strength [59, 64, 129, 130]. Maoxiang Jing [131] implemented additional processing steps to the PE separator to satisfy the requirements of PDOL-based solid-state batteries. Initially, the PE separator was treated with a LiPF_6_ solution to facilitate the loading of initiator for DOL polymerization, following which a high concentration of fast ion conductor Li_6.4_La_3_Zr_1.4_Ta_0.6_O_12_ (LLZTO) solution was applied to one side of the separator. After drying, a PE separator loaded with LLZTO layer (facing lithium metal anode) and initiator was obtained. Direct injection of DOL and LiTFSI into a modified PE separator in cells for in-situ polymerization results in the gradient polymerization of PDOL electrolyte from PE to both sides. The incompletely polymerized gel PDOL can effectively infiltrate the porous cathode, whereas the lithium metal anode side features a highly robust and ion-conductive LLZTO layer that facilitates the uniform deposition of ions and stifles dendrite growth (Fig. 12d). The author draws the stress–strain curve of the electrolyte membrane through tensile testing and found that the fracture strength of the electrolyte is as high as 55.0 MPa.

Conventional PP and PE separators often encounter a challenge of inadequate wettability of DOL, which can be significantly improved by using porous cellulose membranes with high mechanical properties and low cost. Hongfa Xiang [132] utilized PVDF as a binder to load γ-Al_2_O_3_ and aluminum salt initiators onto cellulose paper. The incorporation of γ-Al_2_O_3_ as an inorganic filler significantly enhanced the mechanical strength and electrochemical performance of the resulting polymer. In the process of cell assembly, the modified cellulose paper was directly infused with DOL + LiTFSI solution on both sides. The initiator presents on the cellulose gradually dissolved and triggered the DOL polymerization, and the ensuing gradient diffusion polymerization resulted in improved interfacial contact between the electrode and the electrolyte (Fig. 12e).

Lithium nitrate-containing mesoporous polymer (LP) is an ideal support skeleton for PDOL polymer electrolytes, with its pores filled with in-situ polymerized PDOL [76]. During the cycling, a LiF/Li_3_N-rich interphase layer with high ion conductivity will be formed between the electrolyte and LP, which will improve the stability of Li^+^ transport. The pores of LP provide a dedicated channel for Li^+^ transport, and the electrolyte/LP interface forms a fast Li^+^ transport path, which can effectively improve the ion conductivity of the electrolyte (Fig. 12f). The polymerized PDOL-LP electrolyte membrane exhibits transparency and has a thickness of merely 12 μm. Yet it boasts a high tensile fracture strength of up to 3.5 MPa, which is even 30 times higher than that of conventional PEO-based electrolytes, underscoring its exceptional value as a support skeleton. The researchers fabricated an LFP//Li pouch cell featuring LP-supported PDOL electrolyte and subjected the cell to 1000 bending cycles with a radius of 5 mm. The voltage curves during the bending process exhibited typical voltage behavior and remained unaffected by the bending behavior. Furthermore, the pouch cell could supply power normally in flat, 90° bent, cut, and punctured states, demonstrating excellent performance of LP in terms of toughness and safety. On the other hand, the additional non-conductive support skeleton will occupy a portion of the volume of the conductive polymer, which may lead to a reduction in the ion transport medium.

Augmenting the affinity between the support skeleton and PDOL polymer facilitates the formation of a stable polymer electrolyte membrane. Additionally, a relatively higher porosity can accommodate more conductive polymer, rather than the non-conductive supporting skeleton occupying most of the volume. Jianhua Yan [67] used electrospinning method to prepare a porous polyimide nanofiber (PI-NF) film as a supporting skeleton. First, polyamic acid (PAA) was rendered into a PAA solution and electrospun into PAA-NF at high voltage. Then, the PAA-NF was heated to form a yellow PI-NF film by intramolecular dehydration of the PAA closed-loop molecule. Then, DOL/DME solution containing an initiator and LiTFSI was dropped onto the PI-NF, and the DOL was ring-open polymerized to form a quasi-solid-state electrolyte. The stress–strain curve demonstrated that PI-NF possessed a remarkable tensile strength of 12.3 MPa and a high strain of 89%. The intensive crosslinking between PDOL and PI-NF considerably augmented the tensile strength of the PI quasi solid electrolyte (PIQSE) film to 31 MPa. In comparison with the PP-supported QSE, the PI-supported QSE exhibited superior thermal stability. Specifically, the PIQSE remained structurally intact when subjected to a temperature of 160 ℃, whereas the PPQSE underwent complete melting. Nonetheless, the SE prepared by this method encounter challenges such as complex process procedures.

The support skeleton with ionic conductivity can cooperate with PDOL to simultaneously transport Li^+^, which can improve the overall ionic conductivity of polymer electrolytes. PVDF-HFP is widely used as polymer solid electrolyte because of its high electrochemical stability and ionic conductivity [41, 133–136]. Yulong Xu [123] prepared porous PVDF-HFP membranes using a simple pouring method. SEM images showed that the membrane surface and cross-section were uniformly distributed with a large number of evenly sized pores (with a diameter of about 1 μm). This porous PVDF-HFP has excellent liquid electrolyte retention ability and absorbs a large amount of Li^+^, ensuring sufficient Li^+^ migration ability. Gang Sui [137] further modified the electrospun PVDF-HFP fiber membrane with dopamine (PDA). PDA can form hydrogen bonds with in-situ polymerized PDOL to further enhance the mechanical strength of the electrolyte membrane. Contact angle tests showed that DOL had the best wettability on the PDA/PVDF-HFP skeleton, which is a prerequisite for forming a complete ion transport pathway. Importantly, the tensile strength of the PDOL@PDA/PVDF-HFP electrolyte membrane obtained by these modifications reached 10.1 MPa (152% improvement compared to PVDF), which can meet the requirements of production processes and practical applications.

#### Inorganic Fibers Support Skeleton

**Fig. 13:**
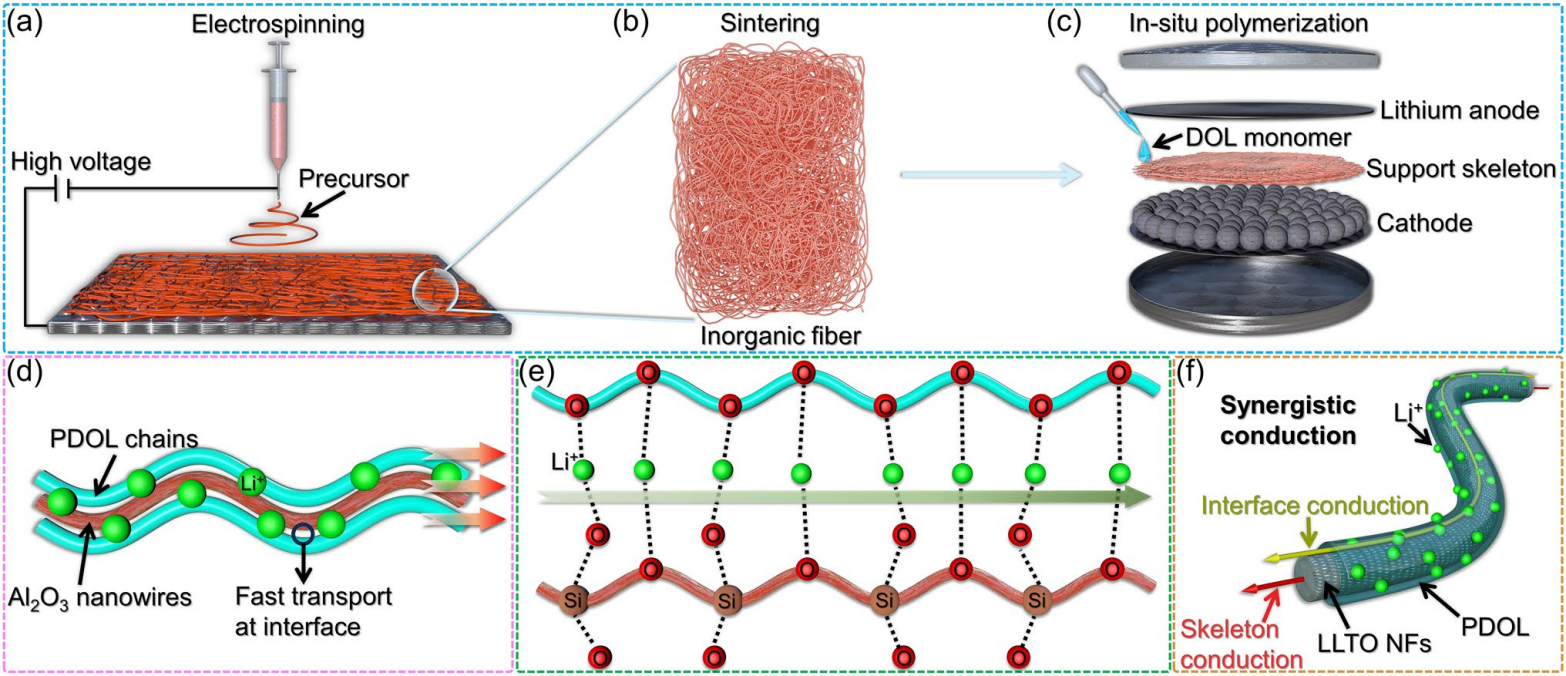
Inorganic fiber materials as PDOL support skeleton. **a** The precursor of inorganic materials is electrospun to prepare a fiber network at high voltage. **b** Inorganic material precursors are sintered to form inorganic fiber support skeletons. **c** Structural diagram of a battery supported by an inorganic fiber skeleton through in-situ polymerization. **d** The interface between Al_2_O_3_ nanowires and PDOL polymer segments constructs a high-speed ion transport channel, accelerating Li^+^ transport [92]. **e** The oxygen atoms of PDOL and the Si–O bonds of SiO_2_ collectively create a highly efficient pathway for Li^+^ migration [139]. **f** LLTO NFs possess Li^+^ transport capability, while the interface between LLTO NFs and PDOL also exhibits high-speed Li^+^ transport capability, the synergistic conduction of both components enhances the overall ionic conductivity of the electrolyte [80]

Inorganic materials possess higher tensile and compressive mechanical strength than polymers, and polymer electrolytes supported by them are expected to have higher stability and safety. Electrospinning is often used to prepare highly porous inorganic membrane materials to accommodate sufficient ion-conductive polymers (Fig. 13a–c) [110, 111, 138]. What is more, the interface between inorganic materials and polymers usually exhibits faster ion transport, and fibrous inorganic materials can guide ion transport and enhance ion transfer efficiency [7]. Moreover, the high thermal stability of inorganic materials can resist thermal decomposition at high temperatures, thereby improving the safety performance of batteries. Therefore, the application of inorganic materials in polymer electrolytes has broad prospects for development.

Shi Wang [92] utilized electrospinning to fabricate an Al_2_O_3_ ceramic fiber skeleton, which was implemented as a supportive matrix for PDOL gel electrolyte. The ceramic fiber membrane constructed a new Li^+^ transmission channel at ceramic/polymer interface (Fig. 13d). The prepared PDOL/Al_2_O_3_ membrane can achieve free-standing and has good toughness. Zhicong Shi [139] prepared a three-dimensional SiO_2_ nanofiber framework as support skeleton by electrospinning. SiO_2_ has better thermal stability, mechanical strength, and electrochemical stability than polymer [140, 141]. The interface between SiO_2_ and PDOL established a swift Li^+^ transmission pathway (Fig. 13e), resulting in a composite electrolyte with an ion conductivity of 1.68 × 10^–4^ S cm^−1^ at 30 °C.

Inorganic materials with inherent Li^+^ transport capabilities as supporting skeletons have two ion conduction channels, bulk ion transport and interface ion transport, thus their ion conductivity has greater advantages [142]. Yue-E Miao [80] prepared a three-dimensional fast ion conductor La_0.56_Li_0.33_TiO_3_ (LLTO) nanofibers (NFs)-supported in-situ polymerized PDOL polymer electrolyte by electrospinning. The porous fibers with a diameter of approximately 500 nm, and the pores are completely filled after in-situ polymerization. In comparison with a pure PDOL membrane, the LLTO-supported electrolyte membrane demonstrates a substantially diminished risk of thermal runaway, and displays self-extinguishing properties during capacitive discharge. Combined with the inherent ion conductivity of LLTO fibers and the interface ion high-speed transmission channel constructed (Fig. 13f), the room temperature ion conductivity of the composite electrolyte was significantly increased to 6.6 × 10^–4^ S cm^−1^, which made a substantial contribution to the application of PDOL-based polymer electrolytes.

The inorganic support skeleton that brings various advantages inevitably reduces the toughness of PDOL polymers, which may limit their application in flexible batteries. Therefore, SE membranes should find a balance point in the long-term conflict between strength and toughness to achieve optimal comprehensive performance. Xueliang Sun [143] referred to “Ceramic-in-Polymer” and “Polymer-in-Ceramic” as representatives of toughness and strength, respectively. A sandwich-structured composite electrolyte was prepared with a high-strength support structure in the middle as “Polymer-in-Ceramic” and high-toughness components on both sides as “Ceramic-in-Polymer” to meet the dual requirements of strength and toughness in the electrolyte. This structural design may be one of the potential solutions in the future to balance the conflicting demands of strength and toughness in polymer electrolytes.

### Inorganic Fillers

Polymer solid electrolytes can often be improved in various aspects such as room temperature ion conductivity, electrochemical stability and mechanical strength through composites with inorganic materials [144]. Numerous relevant studies have confirmed the remarkable impact of lithium fast ion conductors and inert inorganic materials on enhancing the performance of polymer electrolytes [143, 145–149]. The interface between the developed inorganic material surface and PDOL exhibits lower Li^+^ diffusion energy and promotes ion diffusion ability at the interface [150].

**Fig. 14:**
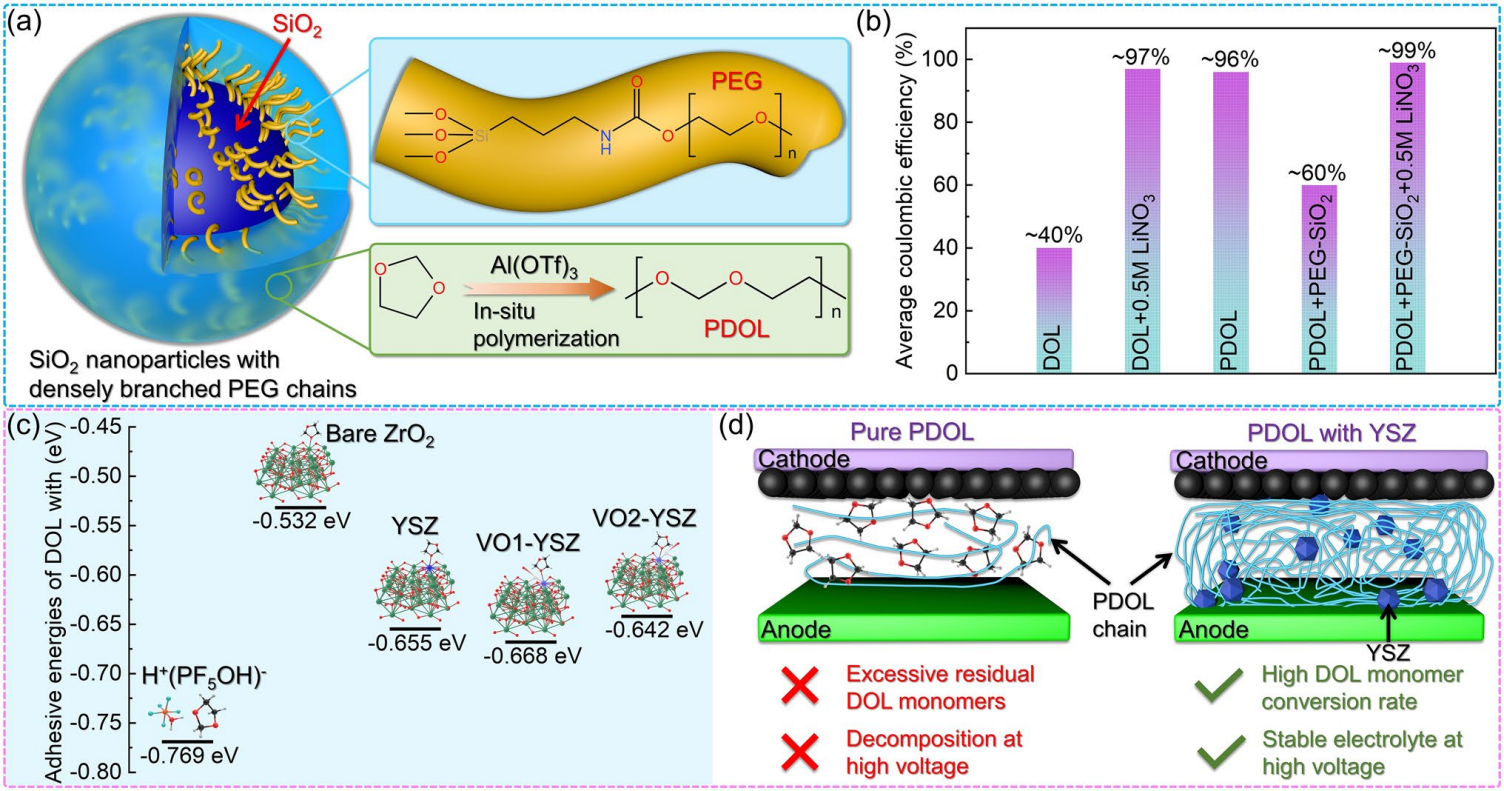
Inorganic materials enhance the performance of PDOL. **a** Schematic diagram of PEG grafting on the surface of SiO_2_ and co-crystallization with PDOL. **b** Average coulombic efficiency for 200 cycles of Li//Cu battery assembled with PDOL-SiO_2_ composite electrolyte [151]. **c** DFT calculated ring-opening binding energy of DOL monomer with initiator, ZrO_2_, zirconium atom on YSZ and oxygen vacancy on YSZ. **d** The auxiliary activation of YSZ substantially enhances the monomer conversion rate of DOL, resulting in a significant improvement in the high voltage capability of the electrolyte, this enhancement enables the attainment of a long service life that aligns with high voltage cathode materials [68]

Lynden A. Archer [151] used aluminum salts to initiate the ring-opening polymerization of DOL in situ to adapt to the electrolyte–electrode interface, and modified it with densely branched PEG chain-nanoparticles of SiO_2_ (Fig. 14a). The nanoscale SiO_2_ can be stably dispersed in the DOL monomer solution, and the high dielectric constant of SiO_2_ can improve the dielectric and flame retardancy of the PDOL-based composite electrolyte. The grafted PEG provides a spatial barrier to prevent filler aggregation, and increases the amorphous region of PDOL by co-crystallizing with PDOL. Not to mention, the interface-grafted PEG also has Li^+^ conduction ability. The synergistic effect of the ion conductivity of the PDOL bulk phase and the interphase improves the overall ion conductivity of the PDOL-SiO_2_ composite electrolyte, reaching 1.5 mS cm^−1^ at room temperature. A Li//Cu battery was constructed and subjected to cycling tests, where the PDOL-SiO_2_ composite electrolyte exhibited an exceptional level of stability with a coulombic efficiency of 99% for 200 cycles (Fig. 14b).

The inability of DOL monomers to withstand high voltage has always been criticized, so electrolytes containing large amounts of DOL monomers often have poor cycling performance in high voltage battery systems, which is one of the most important means to achieve high energy density batteries in the future [152]. Early DOL-based electrolytes could only be used in battery systems below 3.0 V (such as lithium-sulfur batteries) [56, 153, 154], later the polymerization of DOL was carried out to improve the high voltage resistance of PDOL, which can be applied to battery systems around 4.0 V (such as LFP batteries) [59]. Both theoretical and experimental studies have demonstrated that the DOL monomer in PDOL restricts its application in high voltage batteries. Therefore, enhancing the DOL monomer conversion rate during the DOL ring-opening polymerization process is a crucial factor in improving the stability of PDOL electrolytes in high voltage batteries. One of the primary reasons for DOL ring-opening is the presence of a Lewis acid. Yttrium stabilized zirconia (YSZ) is considered a strong Lewis acid materials are highly susceptible to oxygen vacancies and have been reported as effective inorganic fillers for polymer solid electrolytes [49, 150, 155]. Xiangming He [68] prepared PDOL-YSZ composite solid electrolyte by YSZ assisted catalytic DOL ring-opening polymerization. DFT calculations showed that the zirconium atoms and oxygen vacancies on YSZ have similar ring-opening binding energies to the initiator (Fig. 14c), which can effectively promote DOL ring-opening polymerization. The residual rate of DOL monomer in PDOL electrolyte with YSZ addition was only 1.5%, and the decomposition voltage was as high as 5.2 V (Fig. 14d). The assembled high voltage NCM622//Li battery (2.8–4.3 V) maintained a capacity retention rate of 74% after 800 cycles at room temperature at 0.5 C, demonstrating excellent high voltage stability.

Zilong Zhuang [156] synthesized a graphitic-C_3_N_4_ (g-C_3_N_4_)-PDOL composite electrolyte layer in-situ on the lithium metal surface. The flexible PDOL can physically separate the electrolyte from the corrosion of lithium metal, while g-C_3_N_4_ can enhance the ion conductivity of the composite layer, guide ion uniform deposition, and suppress dendrite growth.

## PDOL-Based Electrolytes for Solid-State Lithium Batteries

PDOL-based polymer electrolytes have demonstrated notable enhancements in multiple electrochemical properties through various aforementioned modification methods. Furthermore, it demonstrates exceptional operational stability at ambient temperature in both conventional lithium-ion and lithium-sulfur battery systems. In addition, to broaden its application range, researchers have further enhanced its battery performance, encompassing safety and low-temperature environmental performance, by incorporating functional additives. This advancement has facilitated the practical utilization of PDOL polymer electrolytes in lithium batteries.

### Cycle Performance at Ambient Temperature

We have summarized one of the most important application indicators of some typical reported PDOL-based SSLBs, which is their cycling performance under different cathode materials and operating voltages (Table 2). In early research on DOL and PDOL, due to their poor high voltage resistance, they were mainly used in lithium sulfur battery systems. The most outstanding performance among them is a capacity retention rate of 73.7% after 500 cycles at 0.5 C, and the initial specific capacity also exceeds 1000 mAh g^−1^ [58]. Nevertheless, the practical application of lithium-sulfur batteries encounters notable challenges due to their low operating voltage and areal loading. Consequently, in order to facilitate the widespread utilization of PDOL polymers, it is imperative to enhance their high voltage resistance to achieve compatibility with mainstream high voltage cathode materials.

**Table 2:** Cyclic performance of PDOL polymer electrolyte matched with different cathodes

Battery type	Cathode materials	Initiator	Additive	Voltage range (V)	Initial specific capacity (mAh g^−1^)	Rate (C)	Cycle number	Capacity retention (%)	Refs
Lithium-sulfur batteries	Sulfur	ACNTP	–	1.7–3.0	683	1.0	400	66.5	[56]
	Sulfur	LiPF_6_	DME	1.8–3.0	1005	0.5	500	73.7	[58]
	Sulfur	LiPF_6_	DME/Al_2_O_3_	1.8–3.0	1102	0.1	200	73.0	[59]
	Sulfur	LiFSI	DME	1.7–2.8	910	0.2	300	75.5	[77]
	Sulfur	HFiP	DME	1.8–2.8	1025	0.5	500	68.1	[85]
	Sulfur	LiPF_6_	DME	1.7–2.8	870	0.1	500	45.0	[163]
Lithium-ion batteries	LFP	LiPF_6_	DME	2.5–4.0	137	0.5	700	95.6	[58]
	LFP	LiDFOB	SN	2.5–4.0	127	1.0	1 000	83.55	[75]
	LFP	LiFSI	DME	2.5–3.8	147	1.0	500	69.3	[77]
	LFP	Sc(OTf)_3_	–	2.5–4.0	155	0.5	200	83.5	[91]
	LFP	Al(OTf)_3_	PDA	3–3.75	145	1.0	200	87.1	[137]
	LFP	Y(OTf)_3_	LLZTO	2.8–3.8	135	0.2	200	~ 70.0	[142]
	LFP	LiPF_6_	PI/DME	2.5–3.7	152	0.5	200	91.8	[67]
	LFP	Mg(OTf)_2_	FEC	2.8–4.2	127	1.0	2 000	94.0	[81]
	LFP	LiDFOB	FEC/SN	2.8–4.3	142	2.0	1 000	95.0	[123]
	NCM622	LiPF_6_	DME	2.8–4.3	165	0.1	100	90.9	[58]
	NCM622	Al(OTf)_3_	AlF_3_	2.8–4.2	160	0.5	30	80	[66]
	NCM622	LiPF_6_	–	2.8–4.3	138	0.5	300	85	[129]
	NCM622	LiPF_6_	YSZ	2.8–4.3	165	0.5	800	74	[68]
	NCM523	LiFSI	–	3.0–4.3	136	0.1	100	94	[89]
	NCM811	LiDFOB	FEC/SN	2.8–4.3	145	0.2	100	70.6	[123]
	NCM83	S(C_2_H_4_O_4_)	–	2.8–4.5	205	0.5	100	89.5	[84]
	LCO	LiDFOB	SN	2.8–4.3	140	0.1	60	57.1	[75]
	LMFP	LiBF_4_	TTE	3.0–4.3	130	0.2	100	~ 90	[76]
Sodium-ion batteries	NVP	Al(OTf)_3_	FEC	2.0–3.8	99	5.0	2 000	93.6	[125]

Subsequent investigations have seen researchers employing varied initiators to synthesize PDOL polymer electrolytes with higher DOL monomer conversion rates. This approach has led to improved high voltage resistance and enabled their application compatibility with LFP cathodes. Yang Guanming [91] utilized Sc(OTf)_3_ as the initiator to synthesize PDOL-based electrolyte and achieve a matched SSLB configuration with LFP cathodes. The SSLB underwent 200 charge–discharge cycles within a voltage range of 2.5–4.0 V, demonstrating an impressive capacity retention rate of 83.5%. Moreover, the cycling performance was further enhanced by incorporating additives, including reported compounds such as DME, SN, PDA, Li_6.4_La_3_Zr_1.4_Ta_0.6_O_12_ (LLZTO), PI, FEC, etc. Li Zhenchao [81] achieved the most prolonged cycle life in the reported PDOL system by employing Mg(OTf)_2_ as the initiator for polymerization and incorporating FEC as an additive. Remarkably, after undergoing 2000 charge–discharge cycles at a rate of 1.0 C within the voltage range of 2.8–4.2 V, the PDOL-based electrolyte exhibited an outstanding capacity retention rate of 94%. This remarkable performance highlights its promising application prospects. Under the current conditions of PDOL polymerization process and materials, LFP with relatively low operating voltage has been reported the most as the cathode material for PDOL polymer electrolytes, and it lays the foundation for practical application in terms of cycle life.

In order to meet the demanding energy density requirements of future SSLB, it is imperative for PDOL polymers to satisfy heightened operating voltage criteria. Nonetheless, PDOL encounters substantial challenges when it comes to compatibility with high-voltage cathode materials. Zhao Chen-Zi [66] successfully developed a CEI by incorporating AlF_3_ into the system to inhibit the undesirable reaction between cathode materials and electrolyte. This breakthrough allowed for stable cycling of batteries utilizing PDOL and NCM622 cathode materials. However, the cycling performance, although adequate, did not meet the desired standards for practical applications. Further investigations have confirmed that the lack of high voltage resistance in PDOL systems can be attributed to the interaction between non-polymerized DOL monomers and cathode materials. Therefore, enhancing the conversion rate of DOL monomers is recognized as one of the most effective approaches to improve high voltage resistance capability. He Xiangming [68] successfully synthesized a PDOL solid electrolyte with an impressive DOL monomer conversion rate of up to 98.5% using open environment polymerization and YSZ-assisted initiation. When combined with the NCMM622 cathode material, the PDOL-based SSLB underwent 800 cycles within the voltage range of 2.8–4.3 V, with a remarkable capacity retention rate of 74%. This highly favorable cycling performance stands as one of the best reported for high-voltage PDOL-based SSLB systems, highlighting the potential of PDOL polymers for future application in high energy density SSLBs. In addition, PDOL polymers can also be matched with various cathode materials, including LiNi_0.5_Co_0.2_Mn_0.3_O_2_ (NCM523), LiNi_0.8_Co_0.1_Mn_0.1_O_2_ (NCM811), LiNi_0.83_Co_0.11_Mn_0.06_O_2_ (NCM83), LiCoO_2_ (LCO), LiMn_0.8_Fe_0.2_PO_4_ (LMFP), and even the cathode material Na_3_V_2_(PO_4_)_3_ (NVP) for sodium ion batteries, showing broad application prospects.

In addition, compared with other SPEs that may achieve practical applications such as PEO-based [157], PVDF-based [158], siloxane-based [159], sulfone-based [160], etc., PDOL-based electrolytes demonstrate notable advantages in terms of electrochemical performance and stability. Significantly, PDOL stands out by being prepared through an in-situ liquid–solid transition process, thereby eliminating interphase defects that commonly arise from direct solid–solid contact between solid electrolytes and solid electrodes. A more detailed comparison further unveils a host of compelling advantages. The glass transition temperature of PEO is around 60 °C, which means that its ion conductivity needs to be at high temperatures to meet the application requirements, which is incongruent with the operating environment of the majority of batteries. Additionally, PEO is incompatible with high-voltage cathode materials and unsuitable for integration into high-energy density lithium-ion battery systems [157]. PDOL-based electrolytes can operate normally at room temperature or even low temperatures, meeting the practical application needs of lithium-ion batteries. PDOL-based electrolytes with high monomer conversion rates can match high voltage NCM cathodes, providing higher energy density. The Siloxane-based electrolyte achieved stable 1000 cycles when matched with the LFP cathode, but similar to the problem faced by PEO, its electrochemical stability window is below 4.2 V [159], making it unable to match with higher voltage cathode materials. Both PVDF-based and sulfur-based electrolytes have high-voltage resistance and can be matched with NCM cathodes, indicating promising application prospects. However, PVDF-based electrolytes often require the addition of additional solvents to enhance their ionic conductivity [161, 162], which increases their unsafe factors. Sulfone-based [160] electrolytes are mainly composed of liquid components, and the construction of LiF-rich SEI to protect lithium metal anode achieves excellent long-life cycles. However, their advantages in moving toward high safety, high energy density, and leak free SSLB will lag behind solid electrolytes such as PDOL-based electrolytes.

### Safety and Thermal Stability

There have been numerous explosion and combustion safety accidents caused by thermal runaway in traditional liquid batteries, and solid electrolytes are widely regarded as one of the best solutions to solve this safety hazard in the future [164]. Yet, the decomposition temperature of pure PDOL is relatively low, and it cannot maintain a stable state after being heated. Therefore, there is still much room for improvement in terms of safety.

Chenglin Yan [65] synthesized a thermoresponsive solid electrolyte by copolymerizing DOL with poly(lithium allyl-sulfide) (PLAS), which manifests autonomous reaction with temperature. In the event of a potentially hazardous temperature rise in the battery, the electrolyte will irreversibly block the transport of ions, effectively curbing any further thermal accumulation. The thermoresponsive electrolyte is crosslinked by PDOL and PLAS and exhibits good toughness. When the battery is assembled with the thermoresponsive electrolyte, it is found that the battery can discharge normally and stably before reaching the dangerous temperature. While, in the event of a temperature surge exceeding 70 °C, the battery’s operation is automatically suspended, which effectively eliminates any potential for further temperature escalation and thermal runaway.

Aside from preventing thermal accumulation from the source, flame-retardant PDOL-based electrolytes can also be designed to ensure that no combustion occurs after thermal runaway [84]. Yunhui Huang [83] used TB to initiate the polymerization of DOL to prepare a flame-retardant-type polymer electrolyte. TB produces fluorine radicals as an efficient flame retardant, which can realize a non-flammable electrolyte. The PP separator quickly burns and disappears when heated, while the PDOL-TB electrolyte maintains good non-flammability during heating. When the flame-retardant electrolyte is assembled into a flexible pouch cell, the battery did not experience short-circuiting or burning after being cut twice and can continue to supply power normally. The battery exhibits great advantages in maintaining high safety under sudden destructive conditions.

### Low-Temperature Performance

**Fig. 15:**
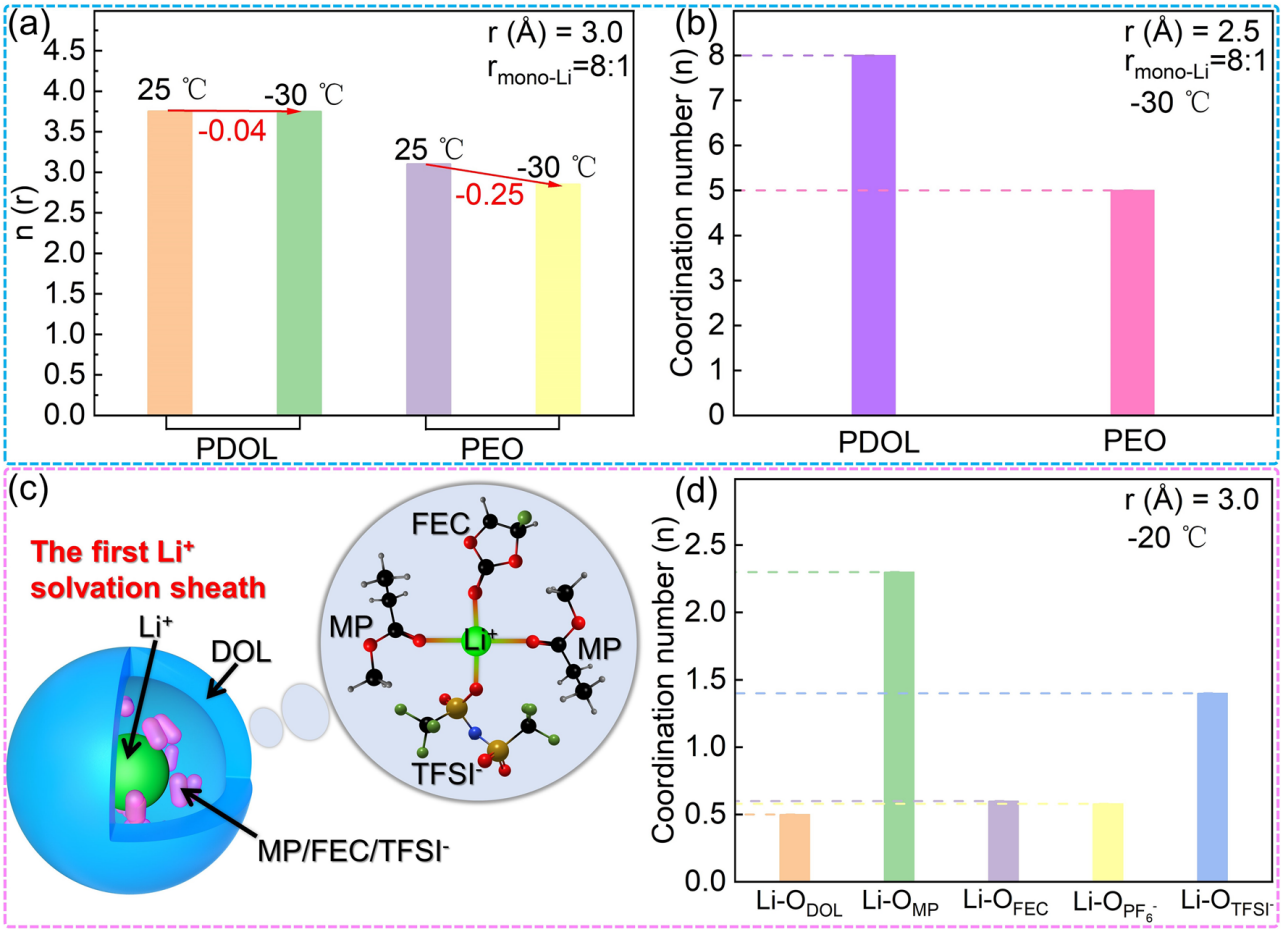
Low temperature performance of PDOL. **a** Li–O integrated RDF of PDOL and PEO (r_mono-Li_ = 8:1). **b** Local coordination number of Li^+^ and O atoms of PDOL and PEO at −30 °C (r_mono-Li_ = 8:1) [166]. **c** The first Li^+^ solvation sheath and snapshot of QSPE from MD simulation for QSPE at -20 °C. **d** The coordination numbers of Li^+^ in the first solvation shell (3 Å) in QSPE at − 20 °C [128]

The ionic conductivity of solid-state electrolytes is one of the biggest challenges that hinders their application, especially at low temperatures where the issue is more severe [165]. Tao Qian [166] compared the ion conductivity of PDOL and PEO at room temperature and low temperature based on MD simulations. The Li–O radial distribution function of PDOL and PEO shows that the coordination number of PDOL is higher than that of PEO within a distance of 3 Å, indicating that Li^+^ in PDOL can contact more oxygen atoms (Fig. 15a). At low temperature (− 30 °C), the coordination number of PEO decreases by 0.25, while that of PDOL only decreases by 0.04 (Fig. 15a). At a temperature of − 30 ℃, PDOL exhibits coordination of 2 Li^+^ with 8 oxygen atoms within a 2.5 Å distance, whereas PEO shows coordination of 2 Li^+^ with only 5 oxygen atoms (Fig. 15b). At high Li^+^ concentration, the advantage of PDOL is even more pronounced. The differential scanning calorimetry (DSC) analysis of liquid electrolyte, PEO and PDOL showed that PDOL has a very low glass transition temperature and no melting temperature, indicating that it has amorphous properties, which is also the reason for its high low-temperature ionic conductivity. Testing has shown that the room temperature ionic conductivity of PDOL reaches 1.2 × 10^–4^ S cm^−1^, which remains at 1.1 × 10^–4^ S cm^−1^ even when reduced to -40 °C. This is much higher than PEO and lays the theoretical foundation for the application of PDOL at low temperatures.

Francesco Ciucci [128] developed a quasi-solid polymer electrolyte (QSPE) based on PDOL and incorporating FEC and MP as plasticizers, which can be cycled at low temperature, and investigated its operating mechanism. The MD simulation snapshot of QSPE is shown in Fig. 15c (within the gray circle), the radial distribution function shows that the first Li^+^ solvated sheath is mainly composed of FEC, MP and TFSI^−^ (Fig. 15c). The MP coordination number of Li^+^ in the first solvated layer is 2.3, and the TFSI^−^ is 1.4 (Fig. 15d). The MP with low viscosity and melting point ensures the Li^+^ diffusion capacity of QSPE at low temperatures. The specific capacity of the NCM811//Li battery assembled with QSPE cycled at 0.2 C for 100 cycles at -20 °C was 109 mAh g^−1^, and the charge–discharge voltage curves at low temperature showed normal Li^+^ intercalation/deintercalation ability without destructive polarization.

### Instability of PDOL Electrolyte

In-situ polymerization of PDOL to form polymer electrolytes often results in the coexistence of DOL monomers due to incomplete conversion. Investigating the decomposition factors of these two components can provide insights into the factors influencing the stability of PDOL-based polymer electrolytes. Perla B. Balbuena [167] used ab initio Molecular Dynamics (AIMD) to simulate the decomposition of DOL monomers on charged Li electrolyte interfaces. Simulation shows that DOL generates (C_2_H_4_)_2_^−^ and OCH_2_O_2_^−^ radicals through a 4-electron mechanism and the cleavage of the double C–O bond. Unfortunately, the previously generated (C_2_H_4_)_2_^−^ will further initiate the continuation of DOL ring-opening and damage to DOL, and as the external electric field increases, the activation barrier decreases, making this reaction easier to proceed. Therefore, the existence of DOL monomer in the high voltage system will affect the electrochemical stability of the electrolyte.

While extensive research has demonstrated the electrochemical stability of PDOL at high voltages, its thermal decomposition mechanism remains unclear. Weidong Zhou [79] analyzed the composition of decomposition gases produced by PDOL electrolyte upon heating to 110 °C using gas chromatography-mass spectrometry (GC–MS). The results showed that the C–O bond of PDOL is prone to breakage at high temperatures, producing formaldehyde and potentially recombining to form dioxane, trioxane, and other cyclic products, and even recombining to form DOL monomers, all of which pose serious threats to the safety of the battery. Therefore, the thermal stability of PDOL-based polymer electrolytes needs to be further improved, which is the foundation of their future applications.

## Perspectives and Conclusions

**Fig. 16:**
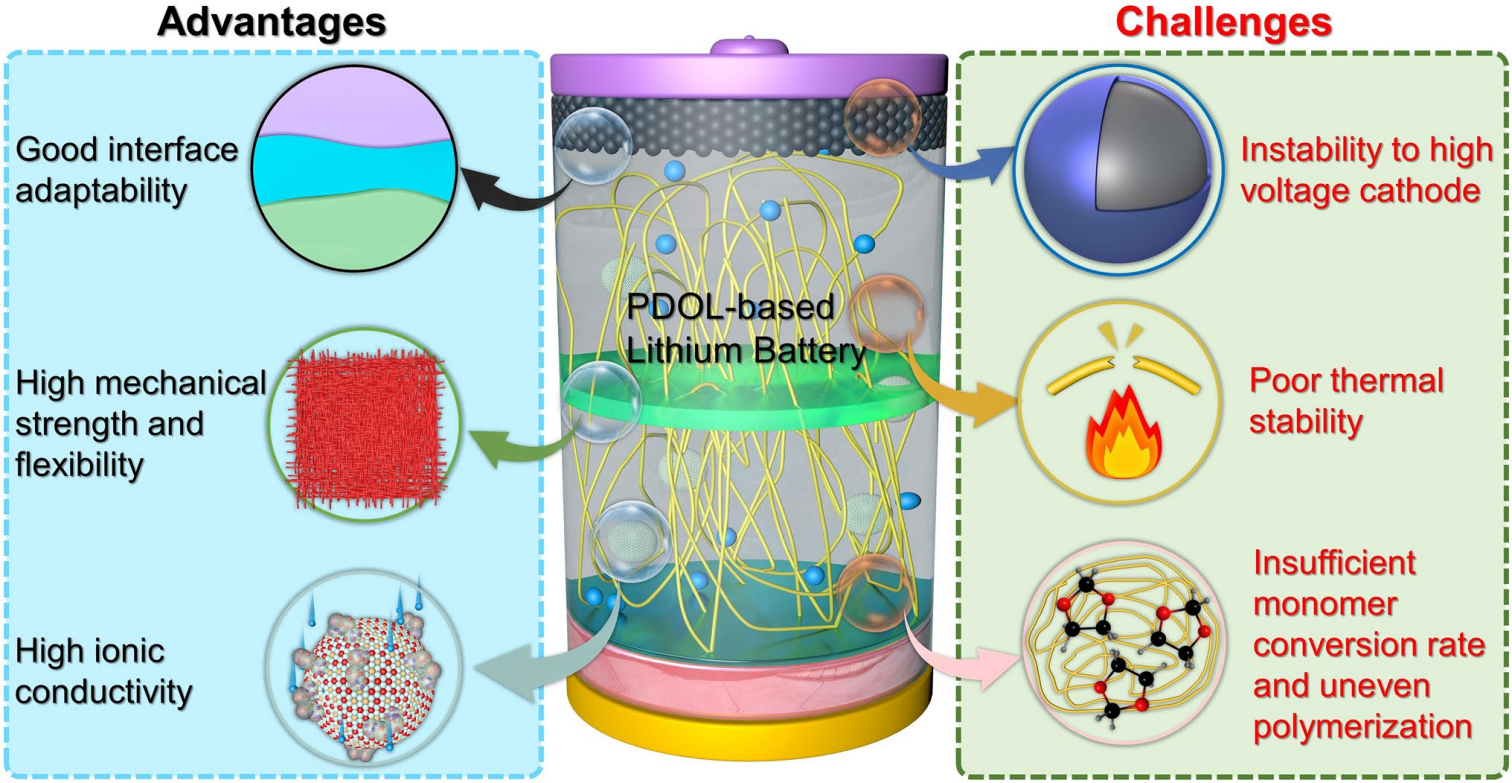
Advantages of PDOL polymer electrolytes and existing challenges in practical applications

PDOL polymer electrolytes have demonstrated numerous advantages over conventional solid-state electrolytes in limited research. These advantages serve as the foundation for their prospective practical applications, primarily encompassing (Fig. 16 left):

1. The electrolyte derived from in-situ polymerization exhibits enhanced adaptability to the electrolyte–electrode interface, thereby facilitating seamless ion transport;
2. Diverse support skeletons significantly enhance the mechanical robustness and flexibility of PDOL polymer electrolytes, enabling them to effectively accommodate external pressure and deformation. This ensures the secure operation of batteries and broadens the application scope of PDOL-based lithium batteries.
3. The addition of plasticizers and inorganic fillers increases the amorphous region of PDOL polymers, thereby yielding heightened ionic conductivity and enabling satisfactory functionality even under exceedingly low temperatures.

Moreover, PDOL polymer electrolytes exhibit good performance in electrochemical stability windows, safety, and Li^+^ transference number. Despite this, its current performance is not yet sufficient for commercial applications. Based on the current reported performance of PDOL-based polymers, we have summarized the remaining challenges and future improvement directions (Fig. 16 right):

1. Unpolymerized DOL monomers are susceptible to decomposition and electrolyte damage upon encountering high voltage cathodes. This can be mitigated by reducing the DOL monomer content or constructing a robust CEI;
2. The thermal stability of PDOL polymer segments is insufficient, rendering them susceptible to decomposition with increasing temperature. To enhance heat resistance, future investigations can focus on reinforcing the strength of chain segments through cross-linking polymerization techniques;
3. In situ polymerization lacks the ability to ensure complete monomer conversion and precise control over the uniformity of polymer distribution. Moving forward, it is imperative to explore more efficient initiators that contain high uniformity of solubility.

Furthermore, one of the future research directions is the modification of the PDOL chain segment itself. Previous studies have demonstrated significant improvement in the electrochemical performance of PDOL by copolymerizing DOL with other monomers to form block or cross-linked polymers [69, 76, 168–170]. The glass transition temperature of block copolymers is frequently dictated by the polymer with the lower temperature [171–174]. Therefore, copolymerizing DOL with other monomers can effectively impede the crystallization of the copolymer and enhance the mobility of the chain segments [69, 169, 170]. Lin Li [168] copolymerized DOL and trioxymethylene as monomers in a ratio of 8/2, resulting in Li^+^ diffusion coefficients of up to 7.37 × 10^–13^ m^2^ s^−1^ and ion conductivity of 3.56 × 10^–4^ S cm^−1^. The product of copolymerization modification of DOL monomer with 1,3-bis(3-glycidoxypropyl)tetramethyldisiloxane has higher thermal stability and wider electrochemical stability window. The silicon containing SEI layer generated during the cycling process can effectively protect the lithium metal anode [170]. Furthermore, the copolymerization products of DOL with trimethylolpropane tris[3-(2-methyl-1-aziridine) propionate] [169], trimethylolpropane triglycidyl ether [76] significantly improved their electrochemical stability, thermal stability, and ionic conductivity. In the future, the addition of functional monomers may bring broader applications to the PDOL chain segment, such as higher mechanical strength, improved safety, and increased ionic conductivity.

To obtain a more comprehensive understanding of the mechanisms underlying PDOL polymer electrolytes, particularly in terms of ion conductivity, electrochemical stability, and other factors, further investigation is warranted regarding their entropy contribution. More work should be carried out (such as concentration, temperature, additives, etc.) to explore the contribution of entropy and the influence on kinetics, product outcomes and controls thereof.

In conclusion, although PDOL solid electrolyte still faces some problems to be solved, the traditional successful polymer electrolyte research methods can be borrowed to improve its performance further. The current monomer polymerization conversion rate of PDOL has reached 98.5%, and can be cycled more than 2000 times when matched with LFP cathode [81], and more than 800 times when matched with high-voltage NCM cathode [68]. This article provides a more specific and in-depth introduction to PDOL-based SPE prepared by in-situ polymerization compared to previous reviews. This review analyzes opportunities and challenges of PDOL electrolytes toward practical application for polymer solid-state lithium batteries in terms of polymerization mechanism, composite modification, and battery applications, and proposes further research prospects for their future potential commercialization. This will provide theoretical guidance and expand the scope of ideas for further research and practical applications of PDOL polymer electrolytes. With the emergence of future materials and the application of novel characterization techniques such as neutron scattering and coherent-THz spectroscopy, the challenges faced by PDOL polymers may be resolved, enabling them to achieve practical commercial applications.
